# Integrated graph measures reveal survival likelihood for buildings in wildfire events

**DOI:** 10.1038/s41598-022-19875-1

**Published:** 2022-09-24

**Authors:** Akshat Chulahwat, Hussam Mahmoud, Santiago Monedero, Francisco Jośe Diez Vizcaíno, Joaquin Ramirez, David Buckley, Adrián Cardil Forradellas

**Affiliations:** 1 Department of Civil and Environmental Engineering, Colorado State University, Colorado, CO 80523 USA; 2 Technosylva Inc., La Jolla, CA USA; 3 Universidad de León, León, Spain; 4 University of Lleida, Lleida, Spain

**Keywords:** Civil engineering, Natural hazards

## Abstract

Wildfire events have resulted in unprecedented social and economic losses worldwide in the last few years. Most studies on reducing wildfire risk to communities focused on modeling wildfire behavior in the wildland to aid in developing fuel reduction and fire suppression strategies. However, minimizing losses in communities and managing risk requires a holistic approach to understanding wildfire behavior that fully integrates the wildland’s characteristics and the built environment’s features. This complete integration is particularly critical for intermixed communities where the wildland and the built environment coalesce. Community-level wildfire behavior that captures the interaction between the wildland and the built environment, which is necessary for predicting structural damage, has not received sufficient attention. Predicting damage to the built environment is essential in understanding and developing fire mitigation strategies to make communities more resilient to wildfire events. In this study, we use integrated concepts from graph theory to establish a relative vulnerability metric capable of quantifying the survival likelihood of individual buildings within a wildfire-affected region. We test the framework by emulating the damage observed in the historic 2018 Camp Fire and the 2020 Glass Fire. We propose two formulations based on graph centralities to evaluate the vulnerability of buildings relative to each other. We then utilize the relative vulnerability values to determine the damage state of individual buildings. Based on a one-to-one comparison of the calculated and observed damages, the maximum predicted building survival accuracy for the two formulations ranged from $58 - 64 \%$ for the historical wildfires tested. From the results, we observe that the modified random walk formulation can better identify nodes that lie at the extremes on the vulnerability scale. In contrast, the modified degree formulation provides better predictions for nodes with mid-range vulnerability values.

## Introduction

Post-fire observations of several historic fires indicated that within affected communities, some ignitable structures tend to survive even when most in close vicinity have been destroyed^1,2^. This apparent spatial randomness in damage patterns raises an important question—can the likelihood of survival of individual buildings be predicted for wildfire events? Fire management agencies and researchers worldwide are utilizing several prominent wildfire behavior models^3–5^. These models are widely accepted and successfully capture wildfires’ behavior in wildlands. However, wildfire propagation mechanisms in communities significantly differ from those in the wildland as additional factors come into play due to the built environment^6^. Previous studies have found that neighborhood and landscape factors contribute noticeably to structures lost in wildfire events^1,2,7–10^. These studies often characterized risk to a structure based primarily on its individual property and fire intensity. Robust computational fluid dynamic models exist for simulating structure-fire interactions that can be used on a community scale^11^. However, their complexity and computational demand limit their practical application. Currently, no generalized community-level framework exists to predict the survivability of structures following a wildfire. Accordingly, there is a need for models to better integrate structures together so those spread mechanisms responsible for structural ignitions, including structure-structure and structure-vegetation interaction, can be accounted for. The mechanism of wildfire propagation shares similarities with the transmission of diseases, which have been extensively modeled using concepts of graph theory^12–15^. Over the years, some studies have looked into the potential application of graph theory for wildfire propagation^6,16–18^ and risk^19^ modeling. These studies were able to capture wildfire behavior both in wildlands and communities successfully. Hence, we can postulate that we can utilize concepts of graph theory to determine the survivability of structures within a fire-affected zone. Building upon the previous work by Mahmoud and Chulahwat^6^ on assessing the vulnerability/risk of the built environment to wildfires, we extend the framework to investigate concepts of graph theory to determine the likelihood of survival of individual structures during wildfire events. Two historic wildfire scenarios from the US are selected for analysis. A graph is formulated for each testbed to model the complex fire interactions between a network of buildings and vegetation. Different centrality measures from graph theory are tested on the two testbeds to determine the relative survivability of each building node by measuring relative vulnerability. Based on the performance of all selected centrality measures, it is observed that none of them provide reasonable accuracy for damage prediction of individual buildings. Therefore, new metrics are proposed to determine building survivability and tested for two historical California wildfires with significant building damage and loss.

## Wildfire graph model

### Graph formulation

Graph theory has found an abundance of applications across different spheres of science, from theoretical research^20–22^ to real-world applications^23–25^. Graphs have become an important tool to model structured populations in approaching various questions related to how a phenomenon, whether information, epidemics, or wildfire, spreads in non-homogeneous populations. Few studies have utilized graph theory to demonstrate its effectiveness in understanding wildfire behavior in the wildland^26,27^. In a previous study by Mahmoud and Chulahwat^6^, a graph model was developed for assessing the vulnerability of ignitable fuels (vegetation and buildings) in communities and considering different heat transfer modes. The framework entailed the formulation of a directed graph ${\mathscr {G}} ({\mathscr {E}}, {\mathscr {V}})$, such that ${\mathscr {E}}$ represented the edges and ${\mathscr {V}}$ the vertices. The vertices defined ignitable fuels within communities, and the edges represented the probability of ignition $P_{tr}^{(i,j)}$ between the individual fuels, calculated based on Eq. (1), where the total probability $P_{tr}^{(i,j)}$ is evaluated by combining individual ignition probabilities from the three primary modes of heat propagation—(1) Convection $P_{conv}^{(i,j)}$ (2) Radiation $P_{rad}^{(i,j)}$, and (3) Ember Spotting $P_{ember}^{(i,j)}$. A detailed description of the individual heat transfer modes is presented in section 1 of the Supplementary Information (SI).

1 $$\begin{aligned} P_{tr}^{(i,j)} = (P_{conv}^{(i,j)} \cup P_{rad}^{(i,j)} \cup P_{ember}^{(i,j)}) \end{aligned}$$

The wildfire graph model^6^ identified ignitable elements that primarily comprise structures and limited vegetation areas within communities. Certain factors, particularly vegetation-related, were not previously considered within the graph framework by Mahmoud and Chulahwat^6^. For instance, vegetation in the vicinity of the wildland-urban interface and within the communities have a noticeable impact on fire behavior^10,28^, but were not considered explicitly in the graph formulation step. In this study, the wildfire graph model is modified to consider missing vegetation and certain building features. The modified graph model is applied to selected testbeds in this study to generate suitable graphs, which are then utilized to determine the survival likelihood of individual buildings within the testbeds.

### Modeling vegetation

**Figure 1:**
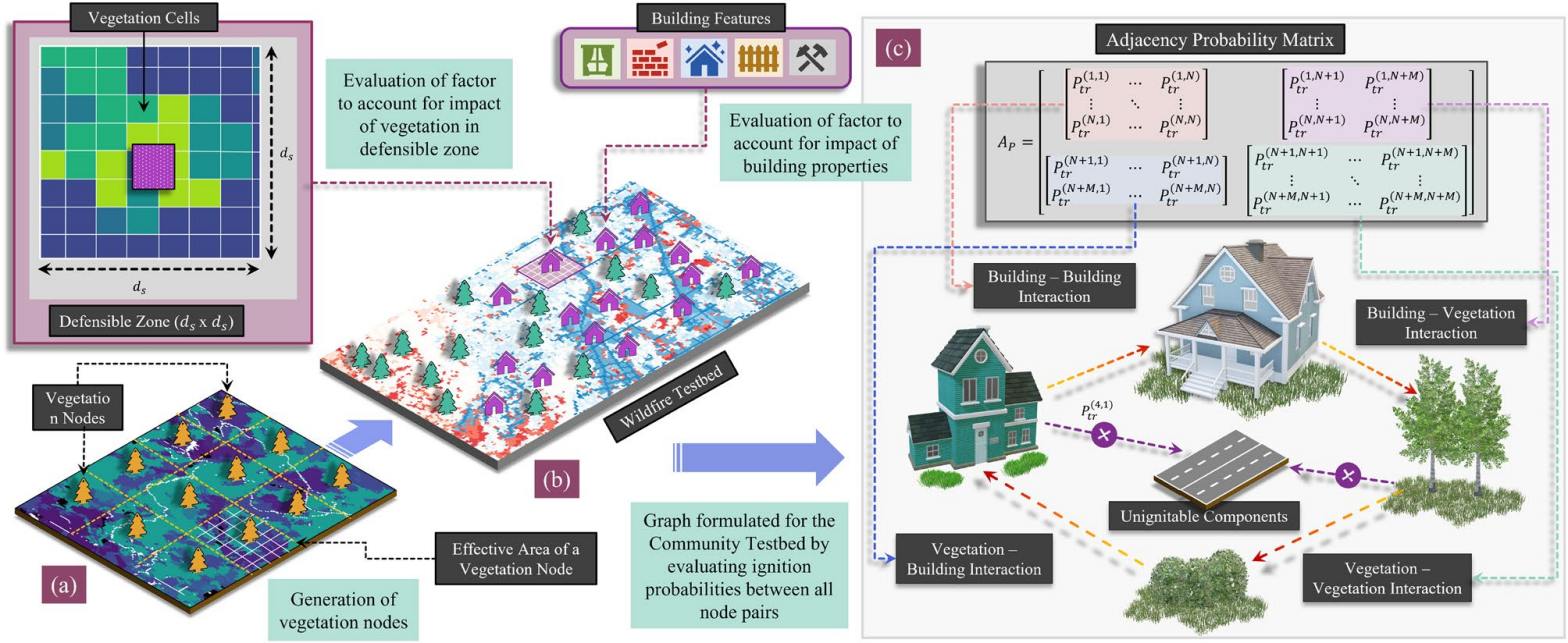
Framework for formulating a graph for a selected wildfire testbed to capture fire interactions between ignitable components within a community. (**a**) Vegetation nodes are formulated from a GIS fuel raster layer (**b**) Building nodes are established for each ignitable structure, and factors are evaluated to account for building properties and vegetation in the defensible zone corresponding to each building node. (**c**) Interactions between all ignitable components (building and vegetation nodes) are realized by formulating an adjacency matrix with edge weights corresponding to the probability of ignition from one node to another.

Vegetation plays a significant role in determining the intensity and rate of spread of a wildfire^1,29,30^. Damage to a community’s built environment is strongly correlated to wildland vegetation at the wildland-urban interface^31,32^, along with the type of vegetation within the defensible space of individual structures^10^. Vegetative fuels can be classified based on proximity to the built environment as—(1) Wildland vegetation and (2) Urban vegetation. The former classification entails vegetation found in dense wildland regions, while the latter relates to vegetation found within or near the confines of urban boundaries that are much more sparse. To incorporate the effects of vegetation within the graph model, we introduce a set of additional nodes ${\mathscr {V}}_n$ to update the graph ${\mathscr {G}}$, such that ${\mathscr {V}} = {\mathscr {V}}_b \cup {\mathscr {V}}_n$, where ${\mathscr {V}}_b$ refers to building nodes and ${\mathscr {V}}_n$ refers to vegetation nodes. The vegetation nodes are generated as a grid of nodes within the domain selected with a uniform spacing $(a_w)$, such that each node represents vegetation over an area $a_w$ x $a_w$. In order to quantify the ignition probabilities from vegetation nodes, we use a GIS fuel raster for a studied area. Sample points are generated within each vegetation node grid cell, as shown in Fig. 1, and for each sampled point, the vegetation type is identified based on the previously presented classification^33^.

A normalized ignition potential score $\eta ^{(k)}$ is calculated for each vegetation node by combining the individual scores of sampled points $\eta ^{(k)}_{i}$, as given by Eq. (2), where $N_{\eta }^{(k)}$ is the total number of sampled points within each vegetation grid cell.

2 $$\begin{aligned} \eta ^{(k)} = \frac{\sum _{k = 1}^{N_{\eta }^{(k)}} \eta ^{(k)}_{i}}{N_{\eta }^{(k)}} \end{aligned}$$

The ignition potential score is utilized in calculating ignition probabilities from vegetation nodes. The graph model includes four types of interactions between the nodes of the formulated graph—(1) Building–Building, (2) Building–Vegetation, (3) Vegetation–Building, and (4) Vegetation–Vegetation. For each type of interaction, the convection $P_{c}^{(i,j)}$, radiation $P_{r}^{(i,j)}$, and ember probabilities $P_{e}^{(i,j)}$ are evaluated and combined to obtain the cumulative ignition probability $P_{tr}^{(i,j)}$. In addition to wildland vegetation, the vegetation within communities, specifically around houses, also plays a major role in the ignition of buildings^1,8,30^. To capture the effect of vegetation in the defensible zone around houses, a similar approach, as described for calculating the ignition potential score of vegetation nodes, is implemented to calculate an ignition factor for individual buildings. An effective distance $d_w$ is selected such that an area $d_w$ × $d_w$ is defined around each building within which the vegetation is considered. The area is divided into a uniform grid such that for each cell, a vegetation score $\eta ^{(k)}_{(i)}$ is sampled. The cumulative ignition factor for each building is then evaluated using Eq. (2), and for each building node *j*, ${\bar{P}}_{tr}^{(i,j)}$ is the updated ignition probability for building $i \in [1,N]$ based on Eq. (3), such that *N* is the total number of building nodes considered in a testbed.

3 $$\begin{aligned} {\bar{P}}_{tr}^{(i,j)} = (1 + \eta ^{(j)}).P_{tr}^{(i,j)} \end{aligned}$$

### Incorporating building features

Studies have shown a positive correlation between vulnerability and building characteristics, ranging from structural to vegetation properties around the buildings^2,9,10,34–36^. In this study, we take into consideration the following building features—(1) Deck Type, (2) Eaves, (3) Roof Type, (4) Vent Type, (5) Fence, and (6) Window Pane. Each feature is further segregated into sub-classifications, as given in Table S1 in the SI. The sub-classification type for each building feature has a noticeable impact on the vulnerability of a building. For instance, a wooden roof or deck is more likely to be ignited than a concrete roof or a deck. To include the effect of building properties in the graph model, we evaluate an ignition factor $i_p^{(i)}$ for each building node *i*, as shown in Eq. (4).

4 $$\begin{aligned} i_p^{(i)} = \sum _{x = 1}^{6} \bigg [ \rho _{(x)} \sum _{y = 1}^{s_{n}^{(x)}} (w_{(y)}^{(x)}.\gamma _{(y)}^{(i)}) \bigg ] \end{aligned}$$

$\gamma _{(y)}^{(i)} \in \{0,1\}$ represents the state of a particular sub-classification for a building feature, such that $\gamma _{(y)}^{(i)} = 1$ represents the presence of a feature and $\gamma _{(y)}^{(i)} = 0$ represents the absence. The variable $w_{(y)}^{(x)}$ is the normalized contribution score for the $y$th sub-classification of the $x$th building feature and weight factor $\rho _{(x)}$ is the normalized contribution score for the $x$th building feature, such that these satisfy the constraints in Eq. (5). They represent the extent to which a particular building feature affects the likelihood of ignition for a particular building. $s_n^{(x)}$ is the number of sub-classifications considered for feature *x*.

5 $$\begin{aligned} \sum _{y=1}^{s_{n}^{(x)}} w_{(y)}^{(x)} = 1 \text {\; and \;} \sum _{x=1}^{6} \rho _{(x)} = 1 \end{aligned}$$

We calculate a cumulative weighted summation for every building node based on the selected building feature classification. This score represents the potential of ignition for each building node due to their respective features. Information regarding features of buildings is derived from the Damage Inspection Database (DINS) by CAL FIRE. The contribution score for each sub-classification $w_{(y)}^{(x)}$ is calculated based on the concept of odds ratios^37^ implemented on the DINS database, and the respective weights are listed in Table S1 of the SI. The weight factor $\rho _{(x)}$ for each feature *x* is assumed to be the same (1/6) in this study based on the assumption that all building features have an equal likelihood of causing the ignition of a structure. The premise is made due to a lack of sufficient data on the contribution of individual building characteristics to the likelihood of building ignition. An important point to note is that the testbeds selected in this study pertain to different communities in the US. For any communities outside of the US, the weight factors would have to be modified since building characteristics can vary significantly depending on location.

## Results

### Graph framework validation

The graph formulation framework was modified to account for vegetation and relevant building features. To confirm the accuracy of these additions to the graph model^6^, we conducted validation tests on Paradise, California (US), which was devastated during the historic 2018 Camp Fire. The Camp Fire started in Butte County in Northern California due to a faulty electric transmission line^38^. In a matter of hours, it reached the community of Paradise to cause significant devastation. In addition to unfavorable wind and climate conditions, high urban and wildland vegetation density in and around residential homes in Paradise fueled the intensity of the wildfire^37,38^.

**Figure 2:**
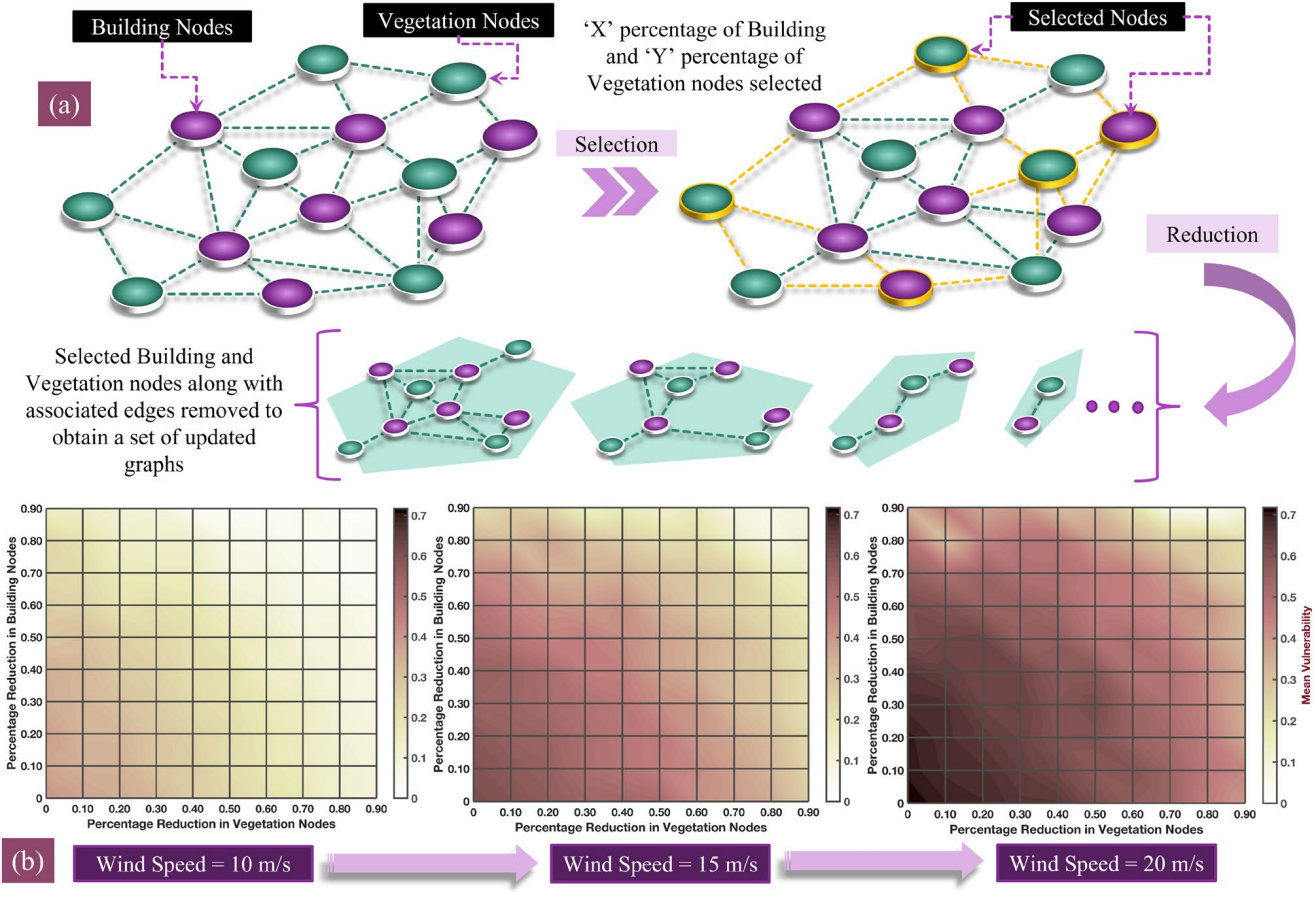
Testing the modified graph model (**a**) Percentage of vegetation and building nodes are altered by selecting nodes at random from each classification. The selected nodes are removed to obtain a set of new graphs. (**b**) The overall community vulnerability is calculated for each modified graph. Heatmaps for the mean vulnerability of Paradise community (Camp Fire) under extreme fire conditions for varying building and vegetation density are shown at different wind speeds.

We conducted a sensitivity analysis to test the efficacy of the graph framework by quantifying the impact of wildland vegetation and buildings along with urban vegetation in the buildings’ proximity to community vulnerability. We introduce two parameters—(1) $v_w \in [0,90]$ and (2) $v_u \in [0,90]$, such that the former represents the percentage reduction in wildland vegetation and the latter represents the reduction in building nodes along with urban vegetation. We model the reduction in wildland vegetation by removing vegetation nodes and the reduction in building nodes and urban vegetation by removing building nodes (see section “Modeling vegetation”). For different permutations of the wildland and building densities, the wildfire graph is first formulated by evaluating the ignition probabilities $P_{tr}^{(i,j)}$ between all node pairs (*i*, *j*). We evaluate each node’s vulnerability by identifying the Most Probable Paths (MPPs) that correspond to paths with the highest probability for fire propagation from an ignited node to a non-ignited one (see “Materials and methods”). We calculate the mean vulnerability of all building nodes to represent the entire community vulnerability. Based on the value selected for the density factors, the $v_w$ percentage of vegetation nodes and $v_u$ percentage of building nodes are selected randomly for removal. We repeat the selection process in a Monte–Carlo simulation to eliminate bias for $K = 100$ iterations. The mean probability is evaluated as the average mean vulnerability of all nodes considered within the testbed for all iterations. Heatmaps are generated for three different wind speeds—10 m/s, 15 m/s, and 20 m/s, indicating the variation in mean vulnerability for different vegetation and building density. As expected, the vulnerability is observed to be maximum for $v_w = 0$ and $v_u = 0$ and minimum for $v_w = 90$ and $v_u = 90$. The pattern of variation observed in the heatmaps, as shown in Fig. 2, is in accordance with expectations.

We also conducted a vulnerability analysis on Paradise to compare the calculated vulnerability patterns under the historic Camp Fire conditions and with the observed damage. A graph for the community of Paradise is created by utilizing pre-fire building and vegetation fuel GIS data for Paradise. A wind speed of 15 m/s with a north-east to south-west direction is assumed, similar to that observed during Camp Fire. Figure S1 in the SI compares the observed damage from the Camp Fire as outlined in a post-fire study conducted by NIST^39^ with the calculated vulnerability. Nodes with high vulnerability values suggest early ignitions compared to other nodes with lower values. The pattern of ignitions observed during the fire coincides with the calculated high vulnerability nodes, suggesting that the graph model framework can capture wildfire interaction with the built environment.

### Node influence metric

To determine the survival likelihood of individual structures within wildfire-affected regions, we borrow concepts from graph theory to assess the relative vulnerability $V_r^{(i)}$ of structures in a wildfire event. The concept of vulnerability can be anchored in the notion of nodal importance, particularly centrality measures^40^, and node influence^41,42^. In the context of graphs, centrality measures are best described as indicators of importance for determining the influence of nodes within a network based on specific criteria. Decades of research have led to significant strides in identifying influential nodes within networks, and the concept has found widespread application in different fields. Some prominent applications of centrality entail the identification of the most influential persons in a social network^22^, super-spreaders of disease^41^, and several others. There are different types of centrality measures in the literature, each effective for specific applications.

In this study, we first evaluate the ability of traditional centrality measures to assess the survival likelihood of buildings. We tested the following widely accepted centrality measures to determine the vulnerability of individual nodes in a graph network—(1) Closeness (Fig. S2 of SI text), (2) Eigenvector (Fig. S3 of SI text), (3) Clustering coefficient (Fig. S4 of SI text), (4) Gravity (Fig. S5 of SI text), (5) Degree (Fig. S6 of SI text), and (6) Betweenness centrality^6^. Each measure was tested on two major wildfires in the US—(1) the 2018 Camp Fire and (2) the 2020 Glass Fire. Both fires are considered among the most destructive fires in the history of California. While in the case of the Camp Fire, high-density urban vegetation around houses resulted in the spread of wildfire, in the case of the Glass Fire, wildland vegetation was the governing factor responsible for wildfire spread. The graph formulated for Camp Fire testbed comprises 11,945 building nodes and 4685 vegetation nodes, while the graph for Glass Fire comprises 3596 building nodes and 15,834 vegetation nodes. Based on the DINS database, 10,923 buildings were damaged during the Camp Fire testbed and 1027 buildings during the Glass Fire. In both cases, similar wind conditions are considered for analysis—wind speed $v_w = 15$ m/s and wind direction $\theta _w = 225^o$, measured counter-clockwise from the x-axis. The vulnerability value calculated for individual nodes is converted to the damage state (see “Damage comparison” section in “Materials and methods”). The calculated damage states of individual nodes are compared to the observed damage states by measuring the prediction accuracy $P_{a}$ (see “Damage comparison” section in “Materials and methods”), which is calculated based on the number of damaged and undamaged nodes. From the results, it is observed that all centrality measures exhibit low maximum prediction accuracy ($\approx 50\%$), but the degree centrality showed slightly higher maximum prediction accuracy ($\approx 55\%$). This is because most centrality measures are not informative for the vast majority of network nodes^42^; instead, they tend to focus on a small number of highly influential nodes, resulting in an underestimation of the spreading power of nodes^43^ (see section 4 in the SI).

A more intuitive approach would be to measure the spreading capacity of nodes to assess the impact of buildings on fire ignitions. Node influence metrics are distinct from centrality measures and explicitly determine highly influential nodes in any network during a spreading process^44,45^. Some approaches to quantify the spreading power of nodes have been proposed in the last decade. One such measure is accessibility^46,47^, which utilizes the concept of random walks to measure how accessible the rest of the network is from a given initiation node. A random walk on a graph can be defined as a random process of sequential selection of nodes and edges to traverse from a particular node on a graph. Another measure is the expected force, developed using concepts of information entropy and random walks, which can assess the strength of spreading power generated by a node^42^. The concept of random walks can be considered relevant in determining the capacity of a node to transmit to other nodes.

### Relative vulnerability metrics

It is noted from the results that all centralities that consider the impact of far-off nodes, like eigenvector, closeness, gravity, and betweenness, appear to be ineffective for the research problem in question, as reflected by their low prediction accuracy. On the other hand, degree centrality, which takes into account the local (or short-range) impact, is observed to perform relatively better (higher prediction accuracy). Degree centrality can be classified into - indegree and outdegree, such that the former refers to the cumulative impact of edges directed towards a node, and the latter refers to the effect of a node on others. In the context of fires, the indegree centrality can be considered a measure of the likelihood of ignition of a node when all its neighbors are ignited, while the outdegree can be regarded as a measure of the capacity of a node to spread fire to its neighboring nodes. It can be hypothesized that the chance of structural ignition is strongly correlated to the ignition of neighboring structures^35^; hence the definition of indegree centrality is well suited for our intended application. In addition, the concept of random walks is related to the spreading power of a node, as it provides the theoretical framework to capture the randomness in the spread of wildfires from one node to its neighboring nodes. Two formulations are proposed in this study to evaluate the relative vulnerability of individual nodes—(1) a modified degree formulation ($V_{md}$) and (2) a modified random walk formulation ($V_{rd}$).

#### Modified degree formulation

**Figure 3:**
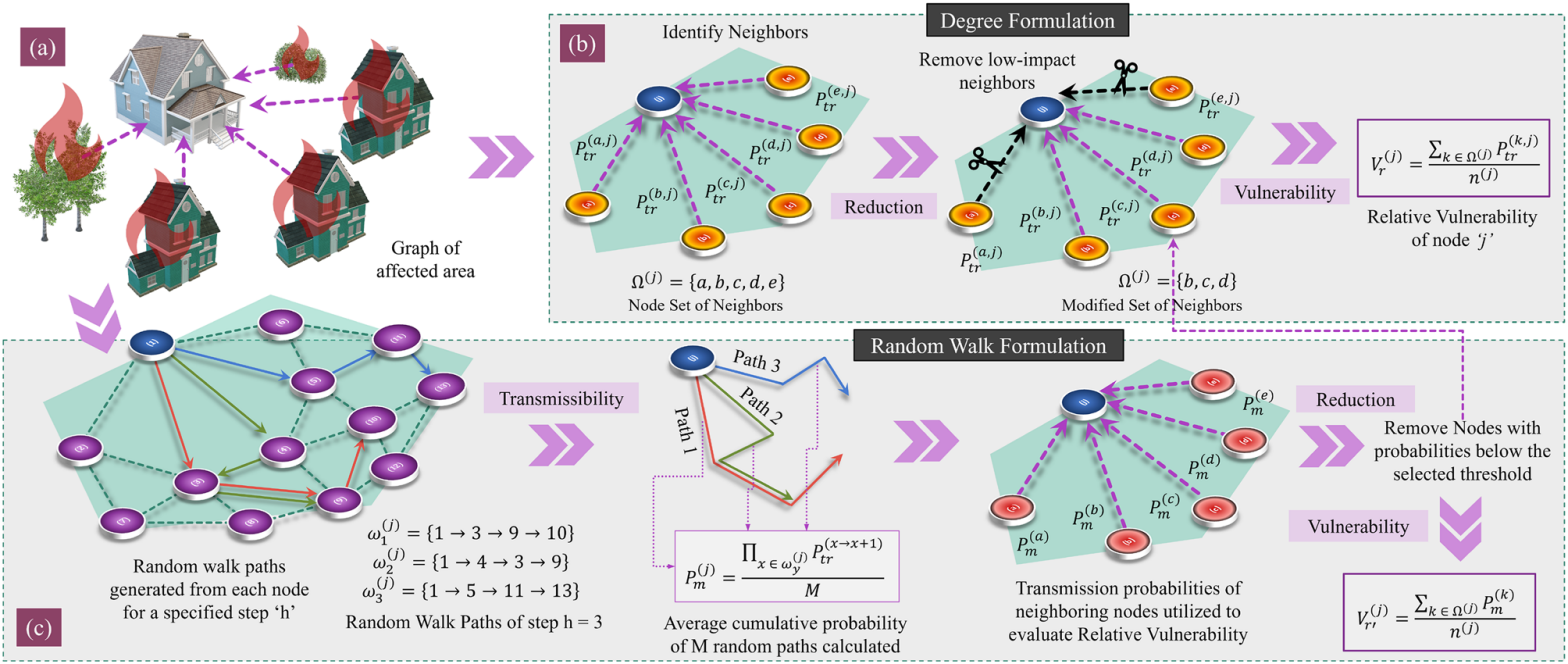
Proposed relative vulnerability framework based on Degree $V_{md}$ and Random walk $V_{mrw}$ concepts implemented on (**a**) formulated graphs of the selected testbeds. (**b**) The modified degree formulation involves the following steps—(1) neighboring nodes identification, (2) Removal of low-impact connections from neighbors, and (3) Relative Vulnerability calculation. (c) The modified random walk formulation includes—(1) Generation of random walks of specific step length for each node, (2) Transmissibility calculation based on random walks generated, (3) neighboring nodes identification, (4) Removal of low transmissibility neighbors, and (5) Relative vulnerability calculation.

The first formulation, modified degree $V_{md}$, is based on the concept of indegree. In this formulation, the relative vulnerability of an individual node is assessed as the mean of incoming edge weights from all neighboring nodes $N^{(i)}$, given by Eq. (6), where $n^{(i)}$ is the number of neighboring nodes. The neighboring nodes to each node *i* are defined by the set of nodes $N^{(i)}$ that has a probability of igniting a target node greater than zero. We introduce an additional constraint to improve the accuracy of the modified degree formulation. In the study by Liu et al.^48^, the authors demonstrated that by removing low-impact links, the spreading ability of each node could be better ascertained. In the context of wildfires, we hypothesize that in most cases, low-impact neighbors do not contribute to structural ignition. Low-impact neighbors are defined as the neighbors with an ignition probability, $P_{tr}^{(N^{(i)},i)}$ towards the target node *i*, below a certain threshold probability $P_{th}$, as shown in Eq. (7). Accordingly, we remove all low-impact (probability) connections between different node-pairs from the graph ${\mathscr {G}}$, such that ${\mathscr {E}}^o = {\mathscr {E}} - \epsilon$, to obtain the modified graph ${\mathscr {G}}^o$, where $\epsilon$ is a set containing all edges with weights below the threshold value. The framework of the modified degree formulation $V_{md}$ is also described in Fig. 3.

6 $$\begin{aligned} V_{md}^{(i)}= & {} \frac{\sum _{k \in N^{(i)}} P_{tr}^{(k,i)}}{n^{(i)}} \end{aligned}$$

7 $$\begin{aligned} P_{tr}^{(N^{(i)},i)}= & {} \Big \{0 | P_{tr}^{(N^{(i)},i)} \le P_{th} \Big \} \end{aligned}$$

We test the modified degree formulation on both testbeds and measure its effectiveness by developing a survivability plot to express the survival likelihood of individual buildings into different vulnerability classes, as discussed in “Materials and methods”. The respective vulnerability map, distribution, and survival plots are shown in Fig. S7 of the SI. The plot represents the survival probability of buildings in each class of the calculated relative vulnerability. The survival probability for the lower class vulnerability values is expected to be higher than for the higher vulnerability classes. Thus, a strictly decreasing curve pattern would suggest a positive correlation between calculated relative vulnerability values and the observed damage states. For the Camp Fire, most buildings were within high-density vegetation; as a result, a higher number of structures were destroyed. While in the case of the Glass Fire, most buildings had sparse vegetation in their surroundings, resulting in relatively lower losses. The impact of removing low-impact connections from the formulated graph is also tested. The survival curves for the two testbeds are shown in the SI in Fig. S9a and c for the case without removal and Fig. S9b and d for the case with removal. From the shape of the survival curves, it can be observed that for the latter case (after removal of low probability links), the survival curves are strictly monotonically decreasing, suggesting a better classification of vulnerability classes. Thus, by removing low-impact connections within the graph, the survival likelihood of individual nodes can be better ascertained. In addition, the prediction accuracy calculated from the vulnerability values (Fig. S8 in the SI) shows maximum accuracy of $57.9\%$ for the Camp Fire and $60.4\%$ for the Glass Fire, which is higher than other node influence metrics tested (Section 4 in the SI).

#### Modified random walk formulation

In the modified random walk formulation $v_{mrw}$, as the initial step, we evaluate the transmissibility $t^{(i)}$ of each node using Eq. (8). A set of $R \in \{r_{(1)}, \ldots , r_{(w)}\}$ random walks are generated for any node *i*, such that $r$th walk for a node is defined as $r_{(w)}^{(i)} = \{ i \xrightarrow {e^{(1)}} v^{(1)} \xrightarrow {e^{(h)}} v^{(h)} \ldots v^{(\lambda )} \}$, where $v^{(h)}$ and $e^{(h)}$ are the node and edge indices at step (*h*) and $\lambda$ is the maximum step size considered for random walk. At each step of the walk $r_{(w)}^{(i)}$, the subsequent node index $v^{(h+1)}$ is determined by selection of one of the neighbors $N^{(v^{(h)})}$ of node $v^{(h)}$ at random.

8 $$\begin{aligned} t^{(i)} = \frac{\sum _{r = 1}^{R} \bigg [\prod _{h \in r_{(r)}^{(i)}} P_{tr}^{(e^{(h)})} \bigg ]}{R} \end{aligned}$$

It can be inferred from observations of post-fire studies that if a building is near fuels with high transmission capacity, the risk of ignition for the building can also be expected to be high^2,30^. In other words, the vulnerability (or survivability) of a node can be considered proportional to the transmissibility $t^{(i)}$ of its neighbors. We define the relative vulnerability as the mean transmissibility of all neighboring nodes, as given by Eq. (9). Similar to the assumption made in the previous formulation, we eliminate the transmissibility values below the threshold value $P_{th}$ to obtain the neighboring node set $N^{(i)}$. The steps involved in the random walk formulation are demonstrated in Fig. 3.

9 $$\begin{aligned} V_{mrw}^{(i)} = \frac{\sum _{k \in N^{(i)}} t^{(k)}}{n^{(i)}} \end{aligned}$$

We test the modified random walk formulation, and the corresponding results for the two testbeds are shown in Fig. S10 of the SI. The prediction results showed maximum accuracy of $57.5\%$ for the Camp Fire and $63.8\%$ for the Glass Fire (Fig. S11 in the SI), which are better than other centrality measures tested (Section 4 in the SI). The survival plots show that the random walk formulation works better for the Glass Fire than the Camp Fire. The random walk overestimates the vulnerability compared with the modified degree formulation. A general observation for the two testbeds is that for most destroyed structures, there are more neighbors with a high probability of ignition than for survived structures. As a result, higher probability neighbors are selected more often for the random walks generated. In this formulation, weak edges (low probability of ignition) are given the same weight as other edges. However, in the case of the modified degree formulation, edges that are higher in number are given more weight. From the results, we observe that the modified random walk formulation can better identify nodes that lie at the extremes on the vulnerability scale. In contrast, the modified degree formulation works better for nodes with mid-range vulnerability values.

**Figure 4:**
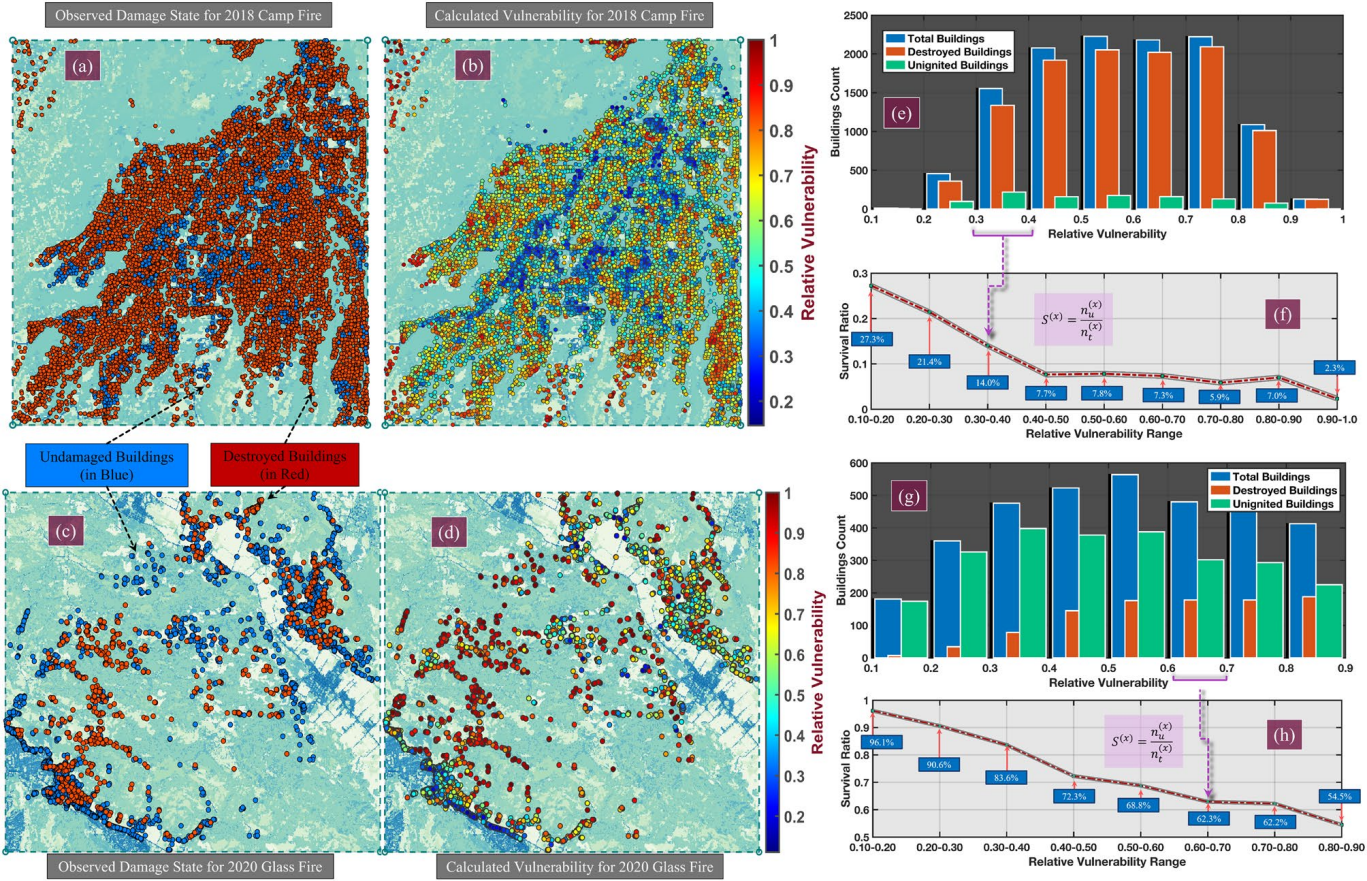
(**a**) Observed damage states of individual structures for Camp Fire. Blue represents undamaged/minimally damaged and red represents significantly damaged structures. (**b**) Relative vulnerability map evaluated for Camp Fire. (**c**) Observed damage states of individual structures for the Glass Fire. (**d**) Relative vulnerability map evaluated for the Glass Fire. (**e**) Distribution of damaged, undamaged and total buildings based on the calculated relative vulnerability for the Camp Fire. (**f**) Survival plot calculated from the distribution plot for the Camp Fire. (**g**) Distribution of damaged, undamaged, and total buildings based on the calculated relative vulnerability for the Glass Fire. (**h**) Survival plot calculated from distribution plot of Glass Fire. All maps were developed in QGIS^49^).

For the modified random walk formulation, the selected step size $\lambda$ has a noticeable impact on the accuracy. To determine the optimal step size, we tested different step sizes ranging from $\lambda = 1$ to $\lambda = 4$ for the two testbeds. Survival curves for different step sizes are shown in Figs. S12 and S13 in the SI. The optimal case is found to be for $\lambda = 1$, as the performance deteriorates with increasing step size. Based on the results from the modified degree formulation, we see that a structure’s survivability strongly depends on the impact of nodes at one degree of separation. As the step size increases, the effect of nodes further away is considered in calculations that create inaccuracies. The graph formulated for wildfire events exhibits high edge density per node; therefore, the distinction between nodal vulnerability diminishes as the step size increases. An underlying assumption made for this formulation is that selection of the next step $v_{(h)}$ in a random walk $r_{(m)}^{(i)}$ from one of the neighboring nodes is based on a uniform distribution. That is to say, each node in the node-set has an equal likelihood of getting selected.

#### Combined results

Based on the results of the modified degree and random walk formulations, it is evident that each formulation has its own advantage and limitation. In a way, the two formulations can be considered complementary to some extent. A combination of the two formulations defined as the weighted average, as shown in Eq. (10), is tested, and the results are shown in Fig. 4. $w_{md}$ is the weight factor for the modified degree formulation, and $w_{mrw}$ is the weight factor for the modified random walk formulation. For all analysis, equal weightage is given to both formulations i.e., $w_{md} = w_{mrw} = 0.50$. The prediction results showed maximum accuracy of $58.15 \%$ for the Camp Fire and $63.15 \%$ for the Glass Fire. For the Camp Fire testbed, an improvement in prediction accuracy is observed for the combined formulation (Fig. S14a in the SI) over the degree and random walk formulations (Figs. S8a and S11a in the SI). While for the Glass Fire testbed, a slight decrease in improvement is observed (Fig. S14b in the SI) over the random walk formulation (Fig. S11b in the SI).

10 $$\begin{aligned} V_{h}^{(i)} = w_{md}.V_{md}^{(i)} + w_{mrw}.V_{mrw}^{(i)} \end{aligned}$$

## Conclusions

The impact of wildfires on communities has been significant, specifically over the last few years. Further high-intensity wildfire events are expected to occur in the future due to a changing climate^50,51^. Accordingly, new tools and methodologies for understanding wildfire behavior are required to aid communities and fire managers in mitigating risk for future events. In this study, we utilized graph theory to simulate fire propagation in communities. We first tested traditional centrality measures to predict the damage states of individual buildings for two historical fire scenarios—the 2018 Camp Fire and the 2020 Glass Fire. Among all the metrics tested, degree centrality showed the highest prediction accuracy. Based on the results, we established two different formulations for calculating the relative vulnerability of individual structures—one based on the concept of degree centrality and the other on the concept of random walks. The two proposed formulations were tested and demonstrated better prediction between calculated and observed damage states than traditional node influence metrics. A difference in accuracy was observed between the two test cases since each community has different characteristics that entail different factors, from the type and density of ignitable fuels to the layout of the community. These different characteristics result in varying degrees of aleatoric uncertainties that cannot be captured in the models, leading to different accuracy for the two cases.

The proposed formulations in this study demonstrated higher accuracy than traditional centrality measures. However, the difference in accuracy was not significantly high due to the complexity of the research problem. For most natural hazards, accurate damage prediction requires a good understanding of how the hazard behaves in a particular scenario and how it interacts with the built environment based on its properties. In general, natural hazards demonstrate higher unpredictability than other physical phenomena^52^, and their chaotic nature^53–56^ makes it challenging to assess their interaction with the built environment accurately. Limited data availability at the community level on internal and external properties of individual structures also hinders accurate damage prediction^57^, more so in the case of wildfires. In the case of wildfires, modeling the interaction between fires and the built environment is still not completely understood. Such modeling requires capturing all factors contributing to ignition at the individual structure level, which is challenging to map out for a given community and computationally expensive to include in a model^7,29,34,58,59^. These factors include fuel tanks in backyards, branches hanging close to homes, and leaves in gutters or on top of roofs. Studies have shown that damage estimation of natural hazards on a local scale is often inaccurate^60^. On the other hand, estimates aggregated over larger scales are more reliable since positive and negative errors balance out^60^. While the accuracy of the proposed formulations for predicting the damage state of every building is not observed to be high, the results indicated that we could capture general damage patterns within different regions by classifying the building nodes into different vulnerability classes.

While the aleatoric uncertainties limit the performance of the proposed model, the accuracy of predictive models can be improved if epistemic uncertainties are considered by better modeling methods^61^. For instance, each test scenario uses a constant wind speed for the entire testbed. High-intensity fires commonly create local weather effects that alter the behavior of local winds^62^. Introducing a computational fluid dynamics (CFD) model to capture wind conditions at a much finer scale should improve the performance of the proposed formulations. Another limitation of the proposed damage prediction model is that the perimeter of the wildfire spread needs to be known beforehand. However, the proposed model can be coupled with other frameworks^6^ that can estimate the extent of fire spread during urban environment events to circumvent this issue. For wildfires, a severe lack of historical data makes using statistical methods, like Machine Learning, a difficult proposition. The development of physics-based predictive frameworks is crucial for better understanding the impacts of wildfire events. The complex mechanisms governing fire behavior, along with the presence of numerous uncertainties, make it challenging to develop computationally efficient models. The focus of this study was to provide some metrics that can determine the survivability of individual buildings within a community for wildfire events with minimal data constraints. Mapping survivability of structures facilitates identifying vulnerable areas within communities or determining effective mitigation strategies by conducting sensitivity analysis.

## Material and methods

The methods utilized in this study are listed below.

### Most probable paths

We define the probability of propagation along a MPP as the product of the edge weights [Eq. (11)], such that ${\mathscr {M}}_{(x)}$ is the set of nodes in *x* MPP given by ${\mathscr {M}}_{(x)} = \{(n_{(1)} \rightarrow n_{(2)}),...,(n_{N_{({\mathscr {M}}_{(x)})}-1} \rightarrow n_{N_{({\mathscr {M}}_{(x)})}})\}$, where $N_{{\mathscr {M}}_{(x)}}$ is the total members in the set ${\mathscr {M}}_{(x)}$. The effective probability from a single ignition source $P_{m}^{(s)}$ is considered as average of *K* MPPs, as given by Eq. (11). Since at any given time multiple ignition sources can be active, we determine the effective vulnerability of any node by evaluating the effect from the most influential ignition node, as given by Eq. (12), where the node set ${\mathscr {S}}$ encompasses all ignition nodes.

11 $$\begin{aligned} P_{m}^{(s)}= & {} \frac{1}{K}\sum _{x=1}^K \big [ \prod _{(i \rightarrow j) \in {\mathscr {M}}_{(x)}} {P_{tr}^{(i,j)}} \big ] \end{aligned}$$

12 $$\begin{aligned} V^{(z)}_{r}= & {} {\max \bigg (P_i^{(s)}.P_{m}^{(s)}\bigg )}_{\{s \in {\mathscr {S}}\}} \end{aligned}$$

To identify MPPs, we utilize a combination of two algorithms (1) Dijkstra’s algorithm^63^ and (2) Yen’s algorithm^64^. The former is used to identify the geodesic path, and the latter to identify *K* geodesic paths in the graph. To utilize these algorithms the weight of edges are modified as $W = log(P_{tr})$, where $P_{tr}$ is the edge weight of original graph ${\mathscr {G}}$. The maximum product problem is converted into a minimum sum problem. Once *K* shortest paths are calculated, the total weight of each path *W* is reverted to obtain the total probability of each MPP.

### Damage comparison

To compare the efficacy of tested formulations, the observed damage state of individual nodes is compared with calculated damage states. The observed damage state of individual nodes for each testbed is obtained from the database developed by CAL FIRE based on post-fire studies conducted. Five damage states are utilized to describe the extent of damage to each building in the DINS database—(1) No damage (2) Affected $(0-10\%)$ (3) Minor $(10-25\%)$ (4) Major $(25-50\%)$, and (5) Destroyed $(>50\%)$. However, in this study, only two classifications are considered—(1) Damaged ($> 10\%$ damage) and (2) Not/Minimally Damaged ($\le 10\%$ damage). The calculated vulnerability values are converted into damage states—(1) Destroyed and (2) Undamaged, based on the relation Eq. (13). $RV_{th}$ is a threshold vulnerability value selected for damage classification.

13 $$\begin{aligned} S^{(i)} = {\left\{ \begin{array}{ll} 0 &{} \hbox { if}\ V_r^{(i)} \le RV_{th} \\ 1 &{} \hbox { if}\ V_r^{(i)} \ge RV_{th} \end{array}\right. } \end{aligned}$$

The calculated damage state $S^{(i)}$ is compared with the observed damage state for each node in the two testbeds. The prediction accuracy $P_{a}$ for each metric is determined as a combination of the number of survived structures predicted with the number of destroyed structures predicted accurately, as given by (14). $N_{cal}^{s}$ and $N_{cal}^{d}$ are the number of survived and damaged buildings that are accurately predicted, $N_{obs}^{s}$ and $N_{obs}^{d}$ are the actual number of survived and damaged buildings observed from the DINS database.

14 $$\begin{aligned} P_{a} = \frac{1}{2} \bigg ( \frac{N_{cal}^{d}}{N_{obs}^{d}} + \frac{N_{cal}^{s}}{N_{obs}^{s}} \bigg ) \end{aligned}$$

### Data background

We used the building footprint database developed by Microsoft, which involves a layer of building footprints mapped from high-quality geospatial satellite images based on deep learning, computer vision, and artificial intelligence techniques. The damage assessment of buildings within and immediately outside the fire perimeter for the selected testbeds was derived from the DINS dataset, which includes location, damage status, and attributes of buildings. Individual attributes data have, for example, roof type, siding material, and the presence of decks and fences. An important point to note is that the Microsoft building database underestimates the number of building footprints. Accordingly, the Microsoft database was updated for this study to account for the missing footprints, and the accuracy of the database was confirmed by comparing it with the DINS dataset. For vegetation data, we utilized surface and canopy fuels, as modeled by Scott and Burgan^33^, in addition to canopy variables such as canopy base height (CBH, m), canopy bulk density (CBD; kg/m^3^), canopy height (CH, m), and canopy cover (CC, %) from LANDFIRE^65^ at 30 m pixel resolution. All maps in this study were developed based on the data sources described in this section by using the QGIS Geographic Information System Software (version 3.22)^49^.

### Survival plot

We categorize the vulnerability values obtained for a testbed into different class intervals, such that (*x*) is a probability class interval defined between the limits $[min^{(x)},max^{(x)}]$. We then define the survival likelihood for each class interval (*x*) using Eq. (15), such that $n_{u}^{(x)}$ is the number of nodes with no/minimal damage and $n_{t}^{(x)}$ is the total number of nodes in a class interval (*x*). The survival likelihood for (*x*) interval determines the probability of a structure surviving if its vulnerability is within that interval. Understandably, the lowest vulnerability interval should exhibit the highest survival likelihood and the highest vulnerability interval vice-versa. For the proposed formulations to accurately predict the relative vulnerabilities of individual nodes, the corresponding survival curve should ideally be strictly monotonically decreasing. Hence, the closest a survival curve is to this pattern, the more likely the formulation will be able to predict the vulnerabilities accurately.

15 $$\begin{aligned} S^{(x)} = \frac{n_{u}^{(x)}}{n_{t}^{(x)}} \end{aligned}$$

## Supplementary Information

Supplementary Information.

## Data Availability

The datasets used and/or analysed during the current study available from the corresponding author on reasonable request.
