# Supplementary material for: Integrated graph measures reveal survival likelihood for buildings in wildfire events
Source: Sci Rep. 2022 Sep 24;12:15954. doi: 10.1038/s41598-022-19875-1 (PMC9509321; doi:10.1038/s41598-022-19875-1)
Supplement: Supplementary file 1 — Supplementary Information. [file 41598_2022_19875_MOESM1_ESM.pdf]

SUPPLEMENTARY INFORMATION

Integrated Graph Measures Reveal Survival Likelihood for Buildings in Wildfire Events

A. Chulahwat et al.

September 13, 2022

1 Graph Formulation

Three primary modes of heat transfer are considered in the wildfire graph model utilized in this study - Convection, Radiation, and Ember Spotting. The model formulates the weights between ignitable nodes in a graph by calculating the probability of ignition from one ignitable node to another based on the wind conditions assumed for the community being assessed.

1.1 Convection

Convection is the transfer of heat by the movement of fluids. In this study, convection corresponds to the ignition of an object due to the direct influence of flames. The probability of convection is defined by Eq. 1, which is unity if the distance between nodes i and j ($d^{(i,j)}$) is within the convection threshold distance ($d^{(i,j)}_{conv}$), and zero otherwise. It is reasonable to assume that if the flames touch an ignitable object, the probability of ignition would be unity.

$$P^{(i,j)}_{conv} = \begin{cases} 1 & \text{if } d^{(i,j)} \leq d^{(i,j)}_{conv} \\ 0 & \text{if } d^{(i,j)} > d^{(i,j)}_{conv} \end{cases} \tag{1}$$

The threshold convection distance is defined as a function of flame height, $h^{(i)}_f$, flame angle, $\theta_f \in [0, 90^o]$, and wind direction $\theta \in [0, 360^o]$. Eq. 2 defines the convection distance model, which includes the effect of uncertainty in wind direction in the form of a wind correlation coefficient $F^{(i,j)}_{cc} \in [0, 1]$, given by Eq. 3. The coefficient attains a maximum value of unity when there is a perfect correlation between the wind direction and the direction of edge from node i to j ($\phi^{(i,j)}$). The wind correlation coefficient is a measure of the uncertainty associated with local changes in wind direction.

$$d^{(i,j)}_{conv} = F^{(i,j)}_{cc}.h^{(i)}_f.tan(\theta_f) \tag{2}$$

$$F^{(i,j)}_{cc} = \begin{cases} cos(|\phi^{(i,j)} - \theta|) & \text{if } |\phi^{(i,j)} - \theta| < 90^o \\ 0 & \text{if } |\phi^{(i,j)} - \theta| \geq 90^o \end{cases} \tag{3}$$

1.2 Radiation

When an object burns, it tends to emit thermal radiation that can cause other nearby objects to catch fire as well. In the case of wildfires, ignitable ways in a community, such as houses, are a major source of thermal radiation. Using the Stefan-Boltzmann law, radiation flux between surfaces of different nodes is calculated. Thermal radiation incident flux on a surface $l \in \mathcal{F}_{(n)} = \{1, .., l, .., N_l\}$ of a way due to a burning surface $k \in \mathcal{F}_{(m)} = \{1, .., k, .., N_k\}$ is calculated by the Stefan-Boltzmann law, where the sets $\mathcal{F}_{(m)}$ and $\mathcal{F}_{(n)}$ represent the surfaces of ways m and n, N_k and N_l are the total number of surfaces of respective ways. The incident thermal radiation flux ($q^{(m,n)}_{(k,l)}$) is defined by Eq. 4, where $A^{(m)}_{(k)}$ is the area of the burning surface, $vf^{(m,n)}_{(k,l)}$ is the view factor from the source to target surface, as given by Eq. 5, σ is the Boltzmann constant, $\epsilon^{(m)}_{(k)}$ is the emissivity of source surface, T_f is the flame temperature and T_a is the temperature of the surroundings.

$$q_{(k,l)}^{(m,n)} = \big(A_{(k)}^{(m)}.vf_{(k,l)}^{(m,n)}.\sigma.\epsilon_{(k)}^{(m)}.((T_f)^4 - (T_a)^4)\big)_{\{k\in\mathcal{F}_{(m)},l\in\mathcal{F}_{(n)}\}} \tag{4}$$

$$vf_{(k,l)}^{(m,n)} = \frac{1}{A_{(k)}^{(m)}} \int_{A_{(l)}^{(n)}} \int_{A_{(k)}^{(m)}} \frac{cos\Theta_{(k)}^{(m)}.cos\Theta_{(l)}^{(n)}}{\pi(d_{(k,l)}^{(m,n)})^2} dA_{(k)}^{(m)}.dA_{(l)}^{(n)} \tag{5}$$

The radiation flux is further utilized to assess the probability of ignition due to radiation mode between the nodes. Detailed description of the procedure is explained in Mahmoud and Chulahwat [7].

1.3 Embers

Of the most prominent modes of propagation are those generated by embers during a wildfire. The embers provide significant complexity to understanding wildfires, as they tend to travel farther downstream than the actual fire front, resulting in multiple fire fronts. The unpredictability and capacity of embers for destruction is a major concern. Ember-driven fire ignitions are heavily influenced by a several factors such as wind direction (θ), wind speed (v_w), and ember size and shape, which are difficult to account for deterministically. The ignition due to embers is modelled using Eq. 6, where $P_{acc}^{(i,j)}$ is the relative probability of access for embers and $g^{(i,j)}(.) : \mathbb{R} \mapsto [0,1]$ is the probability distribution function between nodes i and j, given by a distribution function $S(i, d^{(i,j)}, v_w)$ (Eq. 7). The distribution is uniquely defined for each (i,j) node pair interaction as a function of the volume of source node i ($V_n^{(i)}$), the distance between nodes i and j ($d^{(i,j)}$), and wind speed (v_w). For further details, please refer to the study by Mahmoud and Chulahwat [7].

$$P_{ember}^{(i,j)} = P_{acc}^{(i,j)}.F_{cc}^{(i,j)}.g^{(i,j)} \tag{6}$$

$$g^{(i,j)} = S(V_n^{(i)}, d^{(i,j)}, v_w); \tag{7}$$

Table S1: Odds Ratio calculated from CAL Fire DINS database on 2018 Camp Fire

Index	Feature Classification (x)	Type (y)	$w_{(y)}^{(x)}$
1	Deck or porch on grade	[Composite,Masonry or Concrete,Wood,None]	[0.3,0.3,2.7,2.0]
2	Eaves	[Composite,Masonry or Concrete,Wood,None]	[0.3,0.3,2.7,2.0]
3	Roof	[Enclosed,Unenclosed,None]	[0.3,0.3,2.7,2.0]
4	Vent screen	[Mesh $\leq 4mm$,Mesh $\geq 4mm$,No vents,No screen]	[0.7,1.2,1.1,1.5]
5	Fence	[Combustible,Non-Combustible,None]	[1.8,1.1,0.7]
6	Windows	[Multi-pane,Single-pane]	[0.4,3.0]

2 Building Features

Six building features are considered to incorporate these features' effect on the buildings' vulnerability to wildfires. The features are listed in Table S1, long with their respective feature classification and odds ratio. The odds ratio shows the affinity for each building feature classification. The higher the affinity for a building feature for ignition, the higher the odds ratio assigned. The odds ratio is obtained from the CAL fire DINS database based on the observed damage states of individual buildings. The odds ratio is utilized in Eq. 3 of the main text to evaluate the ignition factor for each building.

3 Graph Model Validation

We used the 2018 Camp Fire to validate the proposed graph vulnerability model. Initial ignition points are chosen based on the post-fire study conducted by researchers at NIST (Maranghides 2021), as shown in Fig. S1. he ignitions point are the ignitions observed at the advent of the Camp Fire when it was about to enter the community of Paradise. A wind direction of north-east to south-west (45° with respect to the negative x-axis) and a constant wind speed of 15 m/s (33.5 mph) is considered based on average wind conditions observed during the fire (Maranghides 2021). A graph is first formulated for the testbed in question by utilizing the graph model with its modifications. For each building node in the graph, a relative vulnerability is calculated based on the concept of Maximum Probability Paths (MPP's), calculated as given by Eq. 8.

Once the weights of each edge ($P_{tr}^{(i,j)}$) are defined for the formulated directed graph, the vulnerability of each way is calculated as the mean probability of most probable paths (MPP) from a particular ignition source (s). The ignition source is defined as the first node in the graph to be activated by fire in wildlands. The position of this node can be on the wildland-urban interface or even inside the community. The probability of propagation along an MPP is defined as the product of the edge weights (Eq. 8), such that $\mathcal{M}_{(x)}$ is the adjacency list of x MPP given by $\mathcal{M}_{(x)} = \{(n_{(1)} \rightarrow n_{(2)}), ..., (n_{N_{(\mathcal{M}_{(x)})}-1} \rightarrow n_{N_{(\mathcal{M}_{(x)})}})\}$, where $N_{\mathcal{M}_{(x)}}$ is the total members in adjacency list $\mathcal{M}_{(x)}$. The mean probability is averaged over K MPPs $P_m^{(s)}$ (Eq. 1), which is selected as $K = 10$ for all analysis due to computational limitations.

$$P_m^{(s)} = \frac{1}{K} \sum_{x=1}^{K} [\prod_{(i \rightarrow j) \in \mathcal{M}_{(x)}} P_{tr}^{(i,j)}] \tag{8}$$

The Relative Vulnerability calculated for each building node is shown in Fig. S1, along with the observed fire incidents reported during the 2018 Camp Fire. The individual fire incidents were derived by researchers at NIST after data-mining social media and other resources, as discussed in the post-fire study (Maranghides 2021).

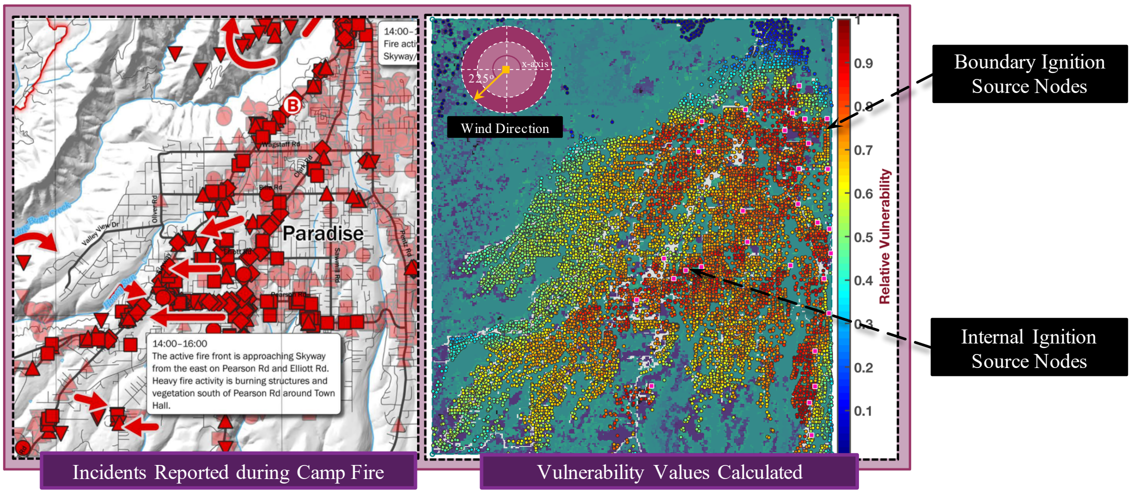

Fig. S1: Vulnerability results calculated based on the modified wildfire graph model, described in the main text, for the 2018 Camp Fire. The figure on the left show the fire incidents reported during the Camp Fire derived based on a study by NIST (Maranghides 2021). The figure on the right shows the vulnerability values calculated for individual building nodes. The Map was developed in QGIS (QGIS Development Team 2022).

4 Node Influence Metrics

For a directed graph $G = (V, E)$ with nodes described by $V = \{v_{(1)}...v_{(N)}\}$, edges described by $E = \{e_{(1)}...e_{(M)}\}$ and the adjacency matrix described by $A = \{a_{(1)}...a_{(N)}\}$, the impact on individual nodes can be estimated by various node influence metrics. The metrics were tested on the two selected testbeds - (1) Camp Fire and (2) Glass Fire, by comparing the calculated damage states with the observed damage states. The calculated vulnerability values $V_r^{(i)}$ are converted into damage states ($S^{(i)}$) - (1) Destroyed and (2) Undamaged, based on the relation Eq. 9. RV_{th} is a threshold vulnerability value selected for damage classification and $V_r^{(i)}$ is the normalized vulnerability calculated based on different node influence metrics calculated at node i. The calculated vulnerability (centrality) values are normalized by the maximum vulnerability value observed in each case.

$$S^{(i)} = \begin{cases} 0 & \text{if } V_r^{(i)} \leq RV_{th} \\ 1 & \text{if } V_r^{(i)} \geq RV_{th} \end{cases} \tag{9}$$

The calculated damage state $S^{(i)}$ is compared with the observed damage state for each node in the two testbeds. The prediction accuracy P_a for each metric is determined as a combination of the number of survived structures predicted with the number of destroyed structures predicted accurately, as given by 10. N_{cal}^s and N_{cal}^d are the number of survived and damaged buildings that are accurately predicted, N_{obs}^s and N_{obs}^d are the actual number of survived and damaged buildings observed from DINS database.

$$P_a = \frac{1}{2}\left(\frac{N_{cal}^d}{N_{obs}^d} + \frac{N_{cal}^s}{N_{obs}^s}\right) \tag{10}$$

4.1 Closeness Centrality

Closeness centrality measures the closeness of a node to other nodes in a graph and can be calculated as the inverse of the sum of the shortest path distances of a node to all other nodes in the graph [1]. However, the impact of nodes at far-off distances can be considered minimal, and their inclusion may lead to inaccuracies in prediction results [5]. Hence, a a modified variation for closeness centrality is utilized such that for each node, the closeness is calculated for nodes within a certain truncation radius R_{th}, as given by Eq. 11. The test results for closeness centrality are shown in Fig. S2.

$$C_{cc}^{(i)} = \sum_{d^{(i,j)} \leq R_{th}, j \neq i} \frac{1}{d^{(i,j)}} \tag{11}$$

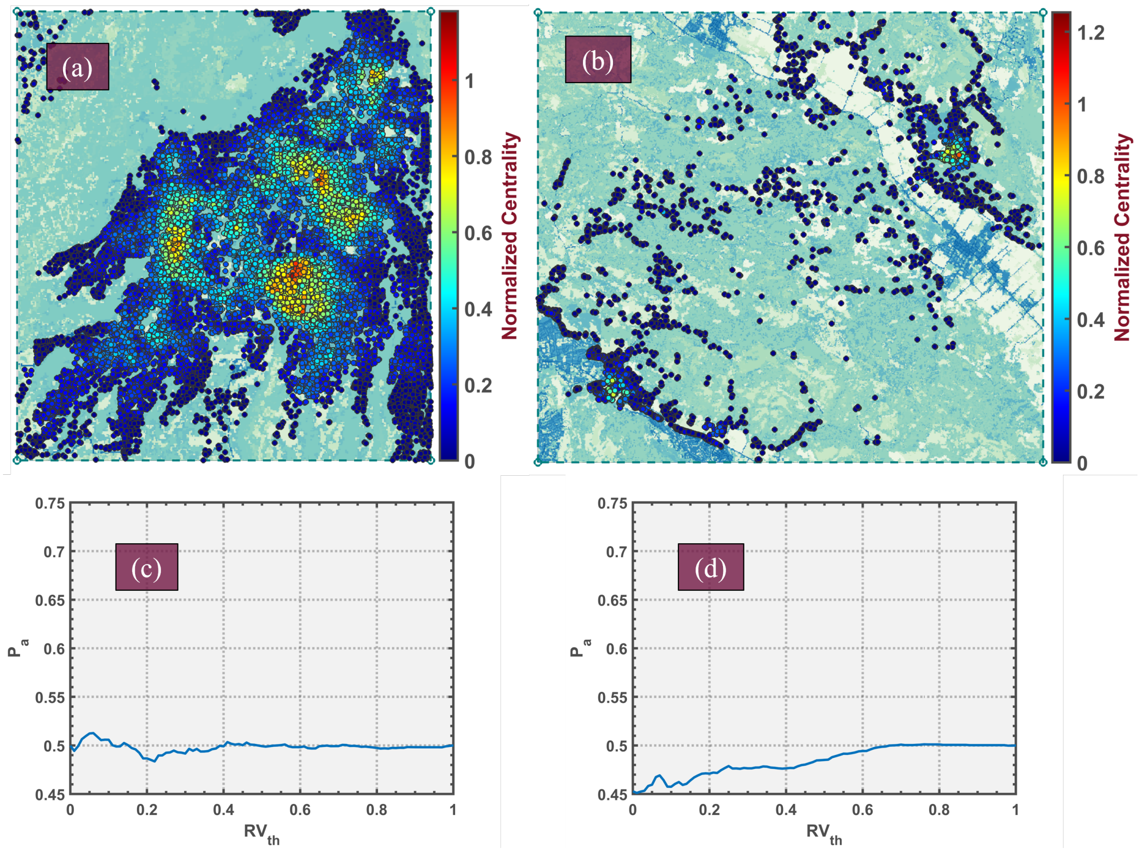

Fig. S2: (a) Closeness centrality values of individual nodes for the Camp Fire. (b) Closeness centrality values of individual nodes forthe Glass Fire. (c) Prediction accuracy P_a for Camp Fire results. (d) Prediction accuracy P_a for the Glass Fire results. Maps developed in QGIS (QGIS Development Team 2022).

4.2 Eigenvector Centrality

Eigenvector Centrality measures the connectedness of nodes in a network. It assigns relative scores nodes based on the concept that connections to high-scoring nodes contribute more to the score of the node in question than equal connections to low-scoring nodes. A high eigenvector score corresponds to connections with high-importance neighbours [3]. The test results for eigenvector centrality are shown in Fig. S3.

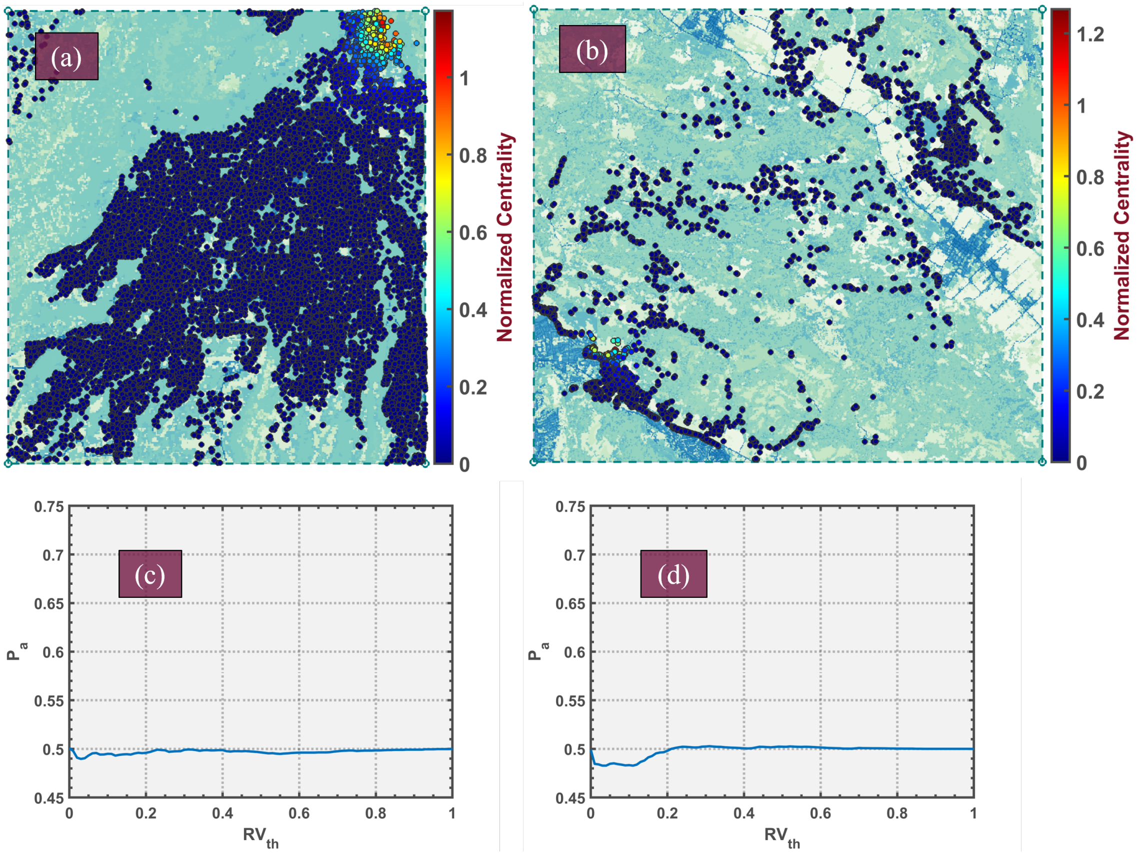

Fig. S3: (a) Eigenvector centrality values of individual nodes for the Camp Fire. (b) Eigenvector centrality values of individual nodes for the Glass Fire. (c) Prediction accuracy P_a for the Camp Fire results. (d) Prediction accuracy P_a for Glass Fire results. All maps were developed in QGIS (QGIS Development Team 2022).

4.3 Clustering Coefficient

The clustering coefficient in graph theory measures the ability of nodes to form clusters (Watts 1998). In the context of wildfires, the clustering coefficient can be utilized to determine the ability of an ignited node to spread fire to its neighbours. The clustering coefficient $C_{cc}^{(i)}$ for a node can be calculated as the proportion of the number of edges between the nodes within its neighbourhood divided by the number of possible edges, as given by Eq. 12. N_i is the neighbourhood of node v_i defined as the list of immediately connected neighbours, and k_i is the total number of neighbours of node i. The results for clustering coefficients are shown below in Fig. S4

$$C_{(i)}^{cc} = \frac{|\{e_{jk} : v_j, v_k \in N_i, e_{jk} \in E\}|}{k_{(i)}(k_{(i)} - 1)} \tag{12}$$

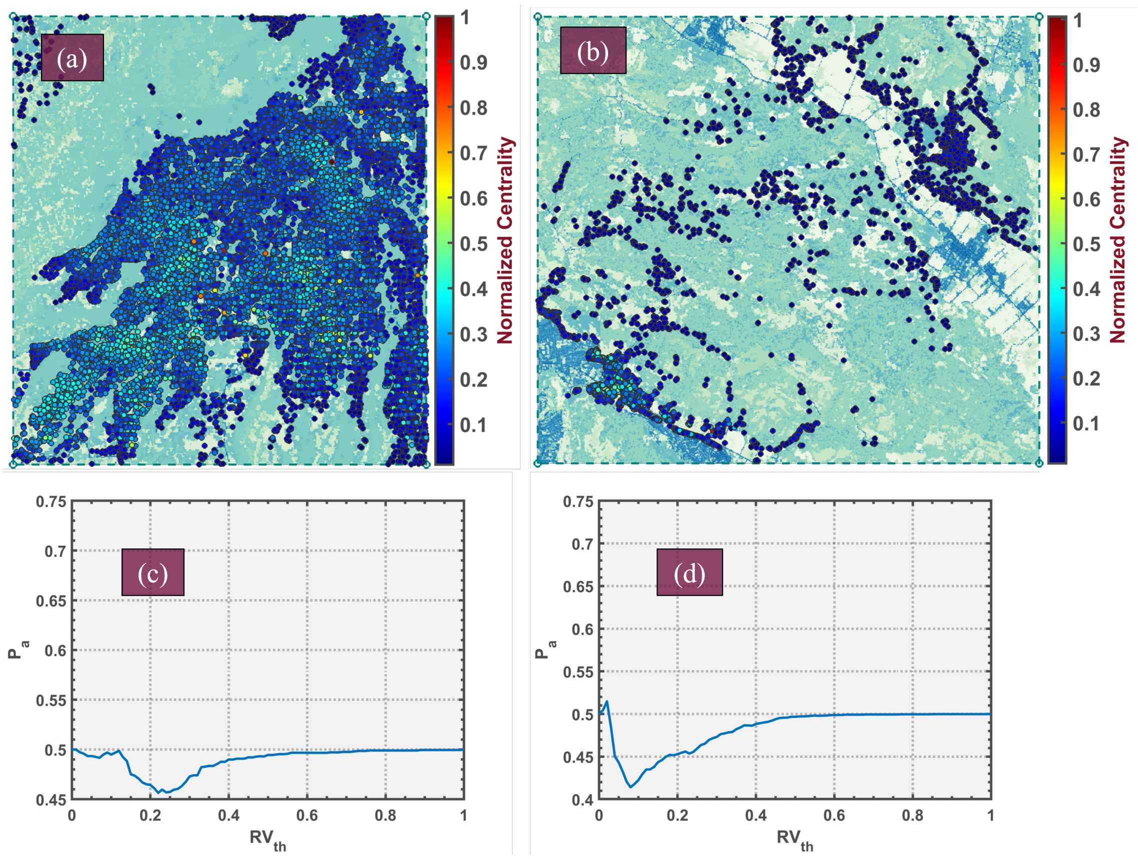

Fig. S4: (a) Clustering coefficient values of individual nodes for the Camp Fire. (b) Clustering coefficient values of individual nodes for the Glass Fire. (c) Prediction accuracy P_a for the Camp Fire results. (d) Prediction accuracy P_a for Glass Fire results. All maps were developed in QGIS (QGIS Development Team 2022).

4.4 Degree Centrality

The degree centrality can be classified into - (1) Indegree and (2) Outdegree. For a directed graph, the indegree ($C_{id}^{(i)}$) for each node i can be calculated as the sum of all edge weights incident on the node (Eq. 13), and outdegree ($C_{od}^{(i)}$) as the sum of all edge weights originating from node i (Eq. 14). $N^{(i)}$ is the set of neighbouring nodes, defined by a degree of separation one from node i. In context of wildfires, the indegree can be defined as the cumulative probability of other neighbouring nodes to cause ignition and the outdegree can be defined as the capacity of a node to transmit fire to its neighbouring nodes. Based on these definitions it can be concluded that the indegree would be more appropriate for the research problem in question. The results for indegree centrality are shown below in Fig. S5.

$$C_{id}^{(i)} = \sum_{k \in N^{(i)}} a^{k,i} \tag{13}$$

$$C_{od}^{(i)} = \sum_{k \in N^{(i)}} a^{i,k} \tag{14}$$

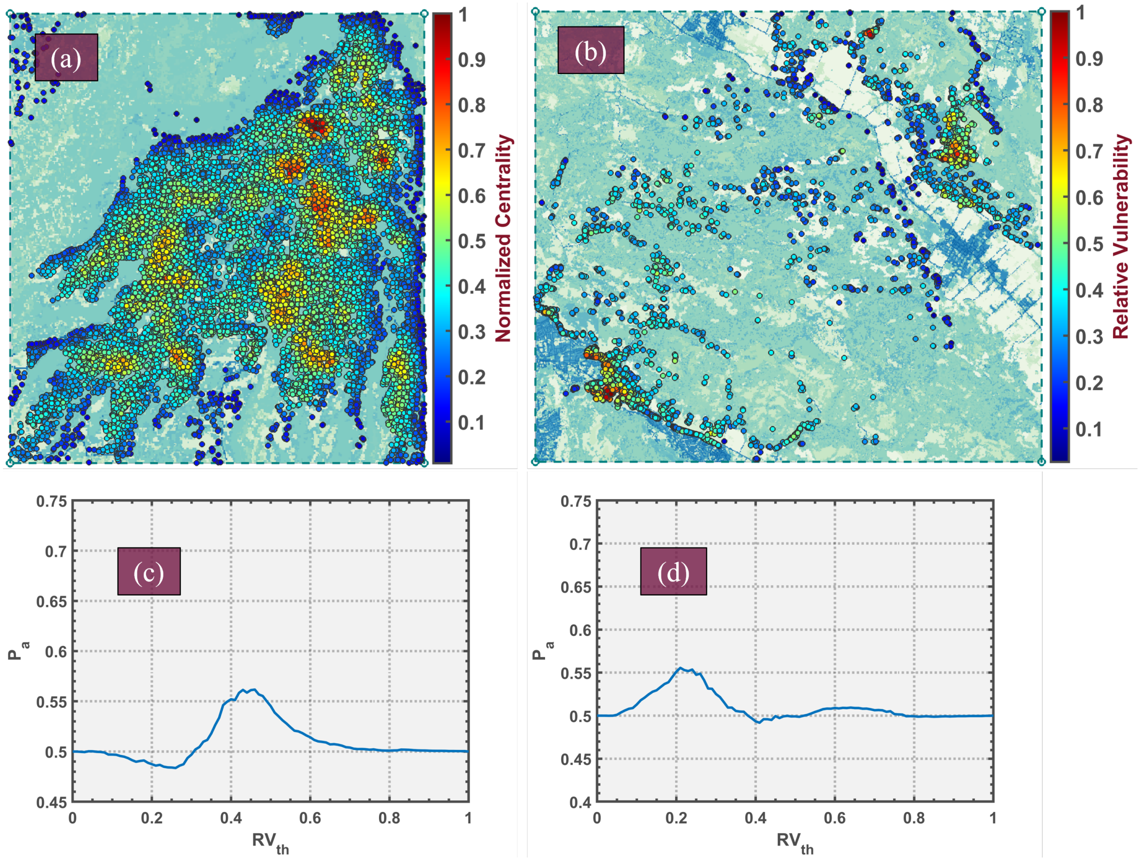

Fig. S5: (a) Indegree centrality values of individual nodes for the Camp Fire. (b) Indegree centrality values of individual nodes for the Glass Fire. (c) Prediction accuracy P_a for the Camp Fire results. (d) Prediction accuracy P_a for the Glass Fire results. All maps were developed in QGIS (QGIS Development Team 2022).

4.5 Gravity Centrality

It is a centrality measured based on the law of gravity that utilizes neighbourhood and path information to determine the influence of nodes [6]. The formulation uses the concepts of Degree and Closeness centralities in combination such that a node with larger degrees (neighbourhood information) and averagely shorter distances to other nodes (path information) are highly influential. The centrality has two limitations - (1) Calculation of shortest paths between all node pairs leads to significant computation time for large-scale networks [4] and (2) Inclusion of impact of far-off nodes can lead to errors due to the accumulation of noise over long distances [2]. In a recent study [5], a variation to the Gravity model was proposed and tested to overcome the mentioned limitations by introducing a truncation radius R_{th}, such that the influence of a node is evaluated based on interaction with other nodes within a certain threshold radius (R_{th}). Eq. 15 shows the formulation for Gravity Centrality of node i such that $d_{(i,j)}$ represents the shortest path distance between nodes i and j and $k_{(i)}, k_{(j)}$ is the degree centrality of nodes i and j. In a wildfire problem, to determine the survivability of node i, we consider the degree of node i ($k_{(i)}$) as Indegree Centrality $C_{(i)^{id}}$ and degree of node j ($k_{(j)}$) as Outdegree Centrality $C^{id}_{(j)}$.

$$C^{gm}_{(i)} = \sum_{d_{(i,j)} \leq R_{th}, j \neq i} \frac{k_{(i)} k_{(j)}}{d_{(i,j)}} \tag{15}$$

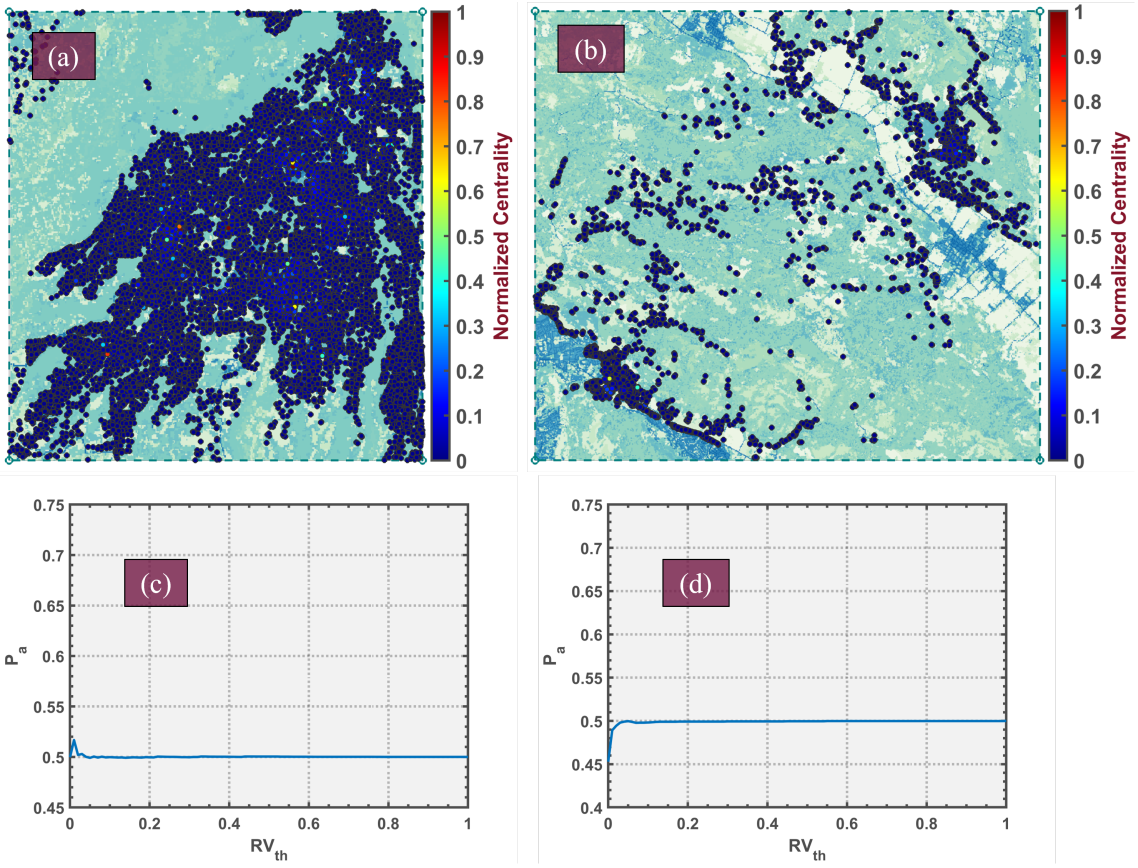

Fig. S6: (a) Gravity centrality values of individual nodes for the Camp Fire. (b) Gravity centrality values of individual nodes for the Glass Fire. (c) Prediction accuracy P_a for the Camp Fire results. (d) Prediction accuracy P_a for the Glass Fire results. All maps were developed in QGIS (QGIS Development Team 2022).

4.6 Betweenness Centrality

Betweenness centrality measures how central a node is to the graph network. it is measured as the ratio between the number of shortest paths between all node pairs in a graph passing through the target node to the total number of node pairs in a graph, as given by Eq. 16. $\sigma^{(s,t)}$ is the total number of shortest paths from node s to node t and $\sigma^{(s,t)}(i)$ is the number of shortest paths passing through node i. For wildfire problems, betweenness centrality has been utilized to determine how important a node is for spreading the wildfire within an area [7], therefore betweenness centrality is not appropriate for determining the survivability of structures.

$$C_{bc}^{(i)} = \sum_{s \neq i \neq t} \frac{\sigma^{(s,t)}(i)}{\sigma^{(s,t)}} \tag{16}$$

5 Degree Formulation

5.1 Results

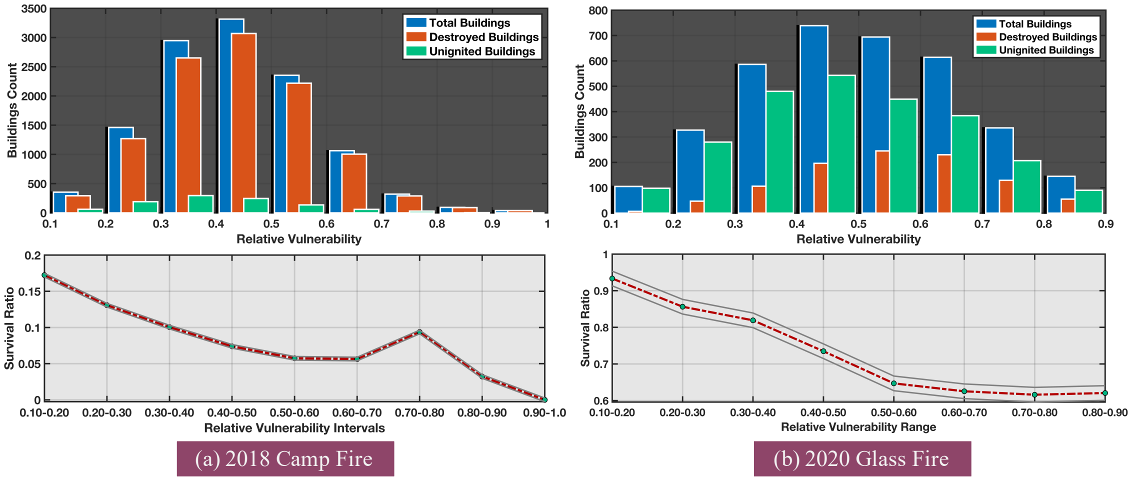

Fig. S7: Distribution of damaged, undamaged, and total buildings based on the calculated Relative Vulnerability from the proposed Degree formulation and Survival plots calculated from distribution plot for (a) the Camp Fire and (b) the Glass Fire.

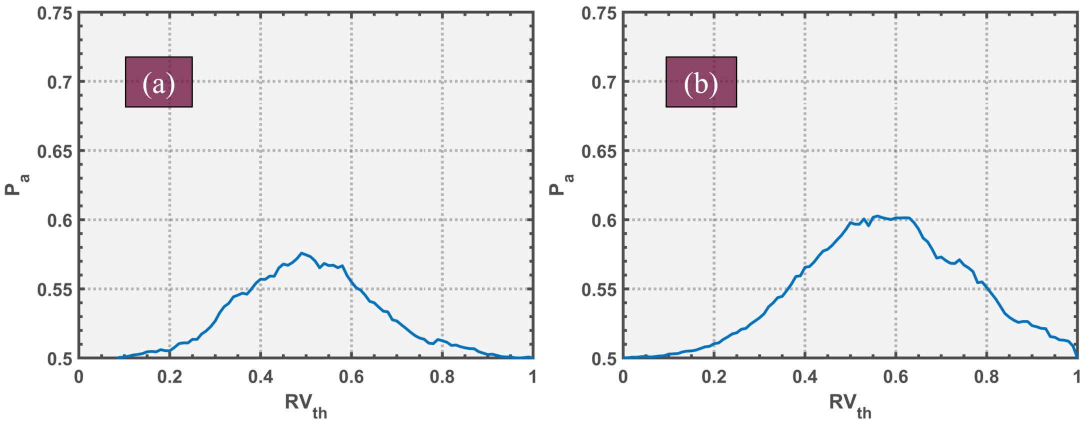

Fig. S8: Degree formulation prediction accuracy results for (a) 2018 Camp Fire and (b) 2020 Glass Fire

5.2 Effect of Node Removal

The graph formulated for each testbed comprises edges corresponding to the probability of ignition from one node to another $(P_t r^{(i,j)})$. ased on the assumption postulated in this study, the impact of low ignition probability nodes is discarded during the Vulnerability calculations. All edges below a certain threshold probability P_{th} are set to zero before calculating the vulnerability of each building node. The threshold ignition probability in this study is assumed to be $P_{th} = 0.25$. Relative vulnerability is calculated for both testbeds with and without the removal of low-impact neighbours, and their corresponding survival curves are shown in Fig. S9. In the case of both testbeds, without the removal of edges, the survival curves do not satisfy the monotonically decreasing criteria. For the formulation to be effective, the survival ratio for low probability intervals has to be higher than the higher probability intervals. Survival curves obtained after removal are quite close to strictly monotonically decreasing functions, suggesting increased effectiveness for the discussed research application.

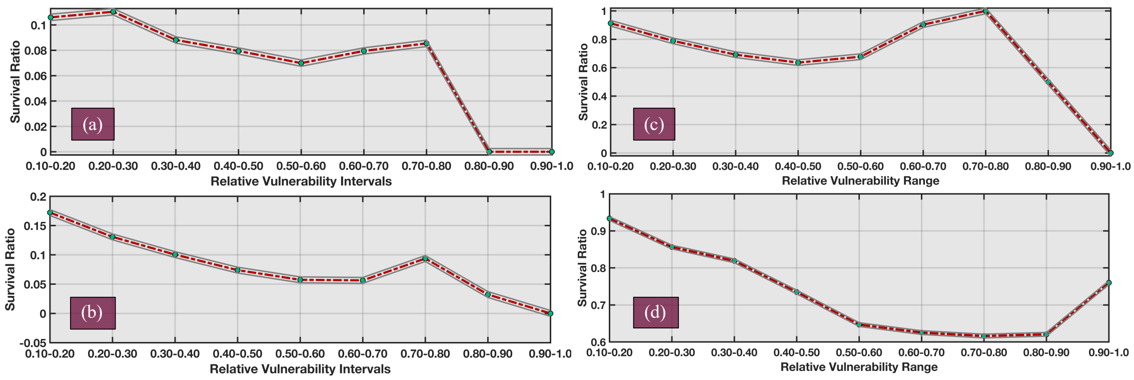

Fig. S9: Survival plots formulated for the (a) Camp Fire without removal of nodes. (b) Glass Fire without removal of nodes. (c) Camp Fire with removal of low-impact nodes. (d) Glass Fire with removal of low-impact nodes.

6 Random Walk Formulation

6.1 Results

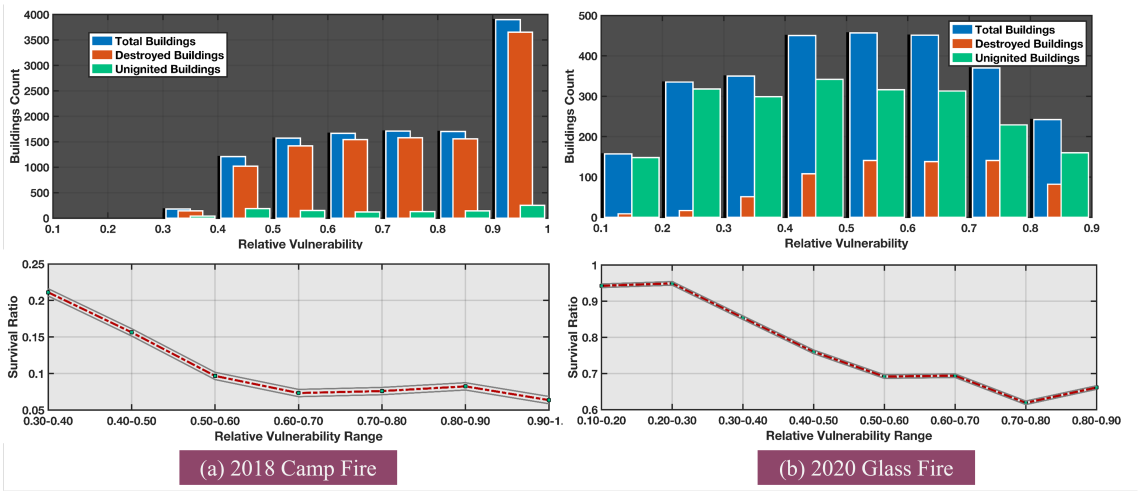

Fig. S10: Distribution of damaged, undamaged and total buildings based on the calculated Relative Vulnerability from the proposed random walk formulation and Survival plots calculated from distribution plot for the (a) Camp Fire and (b) Glass Fire.

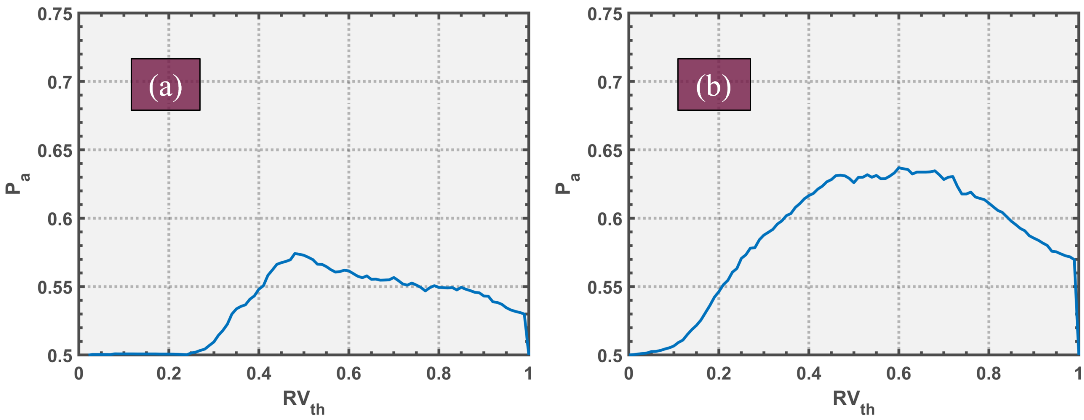

Fig. S11: Random Walk formulation prediction accuracy results for (a) 2018 Camp Fire and (b) 2020 Glass Fire.

6.2 Effect of Step-Size

The Random Walk formulation is tested to evaluate the effect of step-size on its accuracy. The step size is varied from $1-4$, and the corresponding probability distribution and survival curve are developed to assess the performance. The curves for Camp Fire testbed are shown in Fig. S12 and for the Glass Fire in Fig. S13. From the results, it is evident that as the step size increases, the diversity in probability distribution starts to narrow down, which is unsuitable. In addition, the survival curves also tend to deter from their expected behavior (monotonically decreasing). This reaffirms the hypothesis postulated in the main text that the survivability of any building is highly correlated to the impact of immediate neighbours.

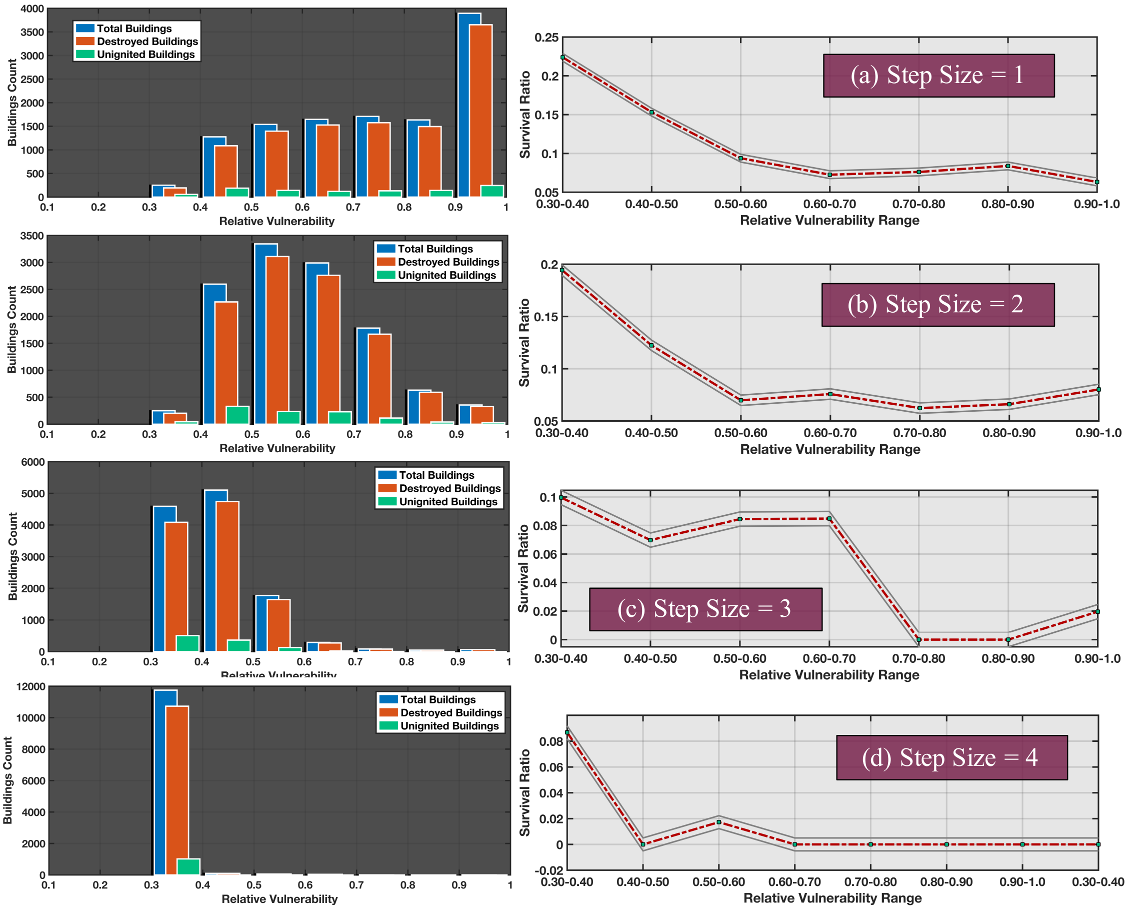

Fig. S12: Probability distribution curves obtained from the random walk formulation and the corresponding survival plots for a step size ranging from $1-4$ for the Camp Fire.

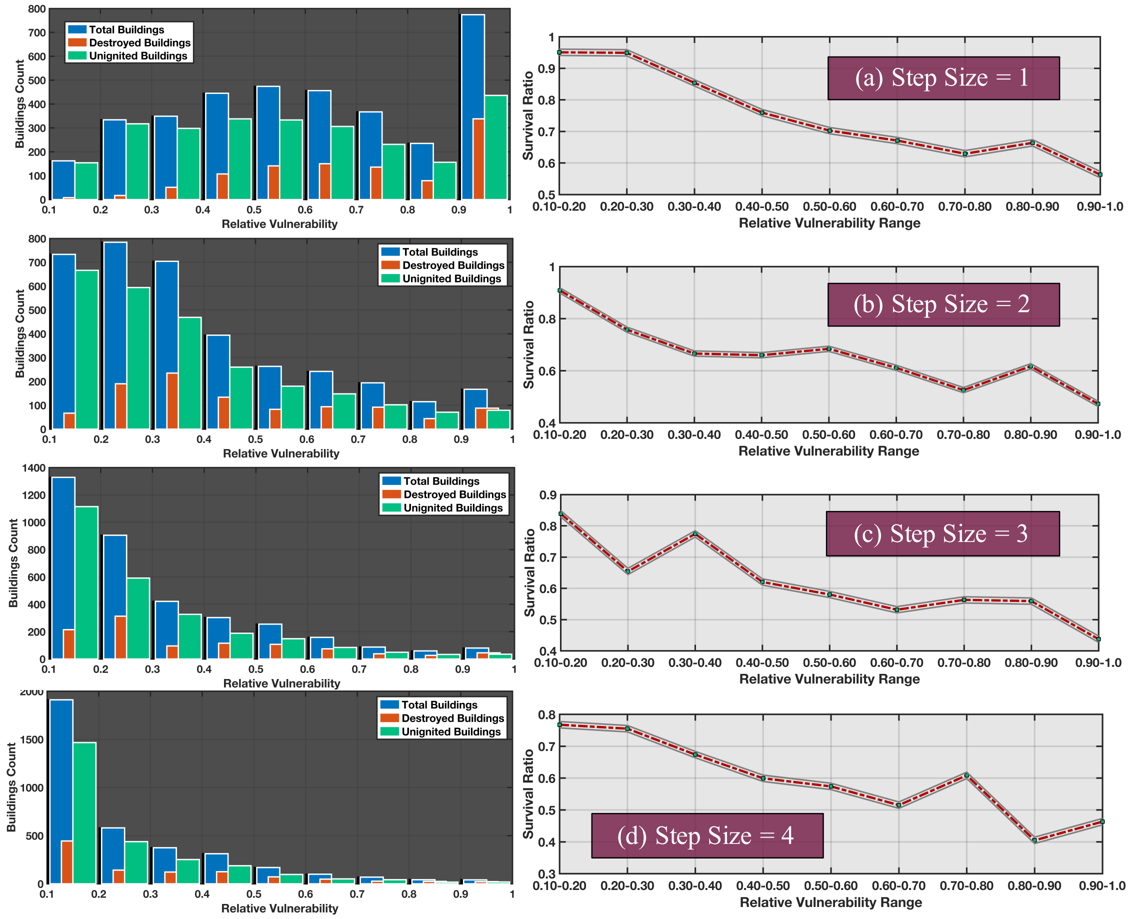

Fig. S13: Probability distribution curves obtained from the random walk formulation and the corresponding survival plots for a step size ranging from $1-4$ for the Glass Fire testbed.

7 Combined Formulation

7.1 Accuracy Results

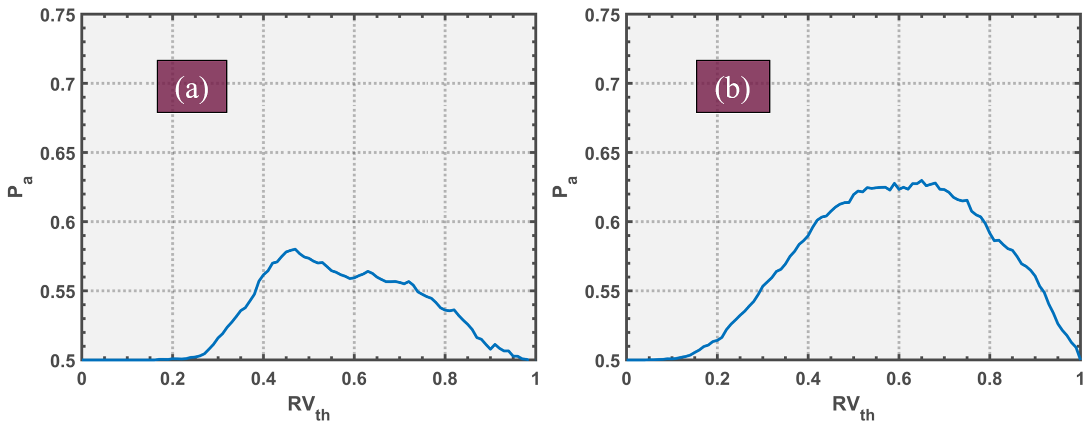

Fig. S14: Combined formulation prediction accuracy results for the (a) Camp Fire and (b) Glass Fire.

References

[1] A. Bavelas. Communication patterns in task [U+2010] oriented groups. *Journal of the Acoustical Society of America*, 22(6):725–730, 1950.

[2] D. Chen, L. Lü, M. S. Shang, Y. C. Zhang, and T. Zhou. Identifying influential nodes in complex networks. *Physica A*, 391:1777–1787, 2012.

[3] F. S. Christian and et al. Eigenvector centrality for characterization of protein allosteric pathways. *Proceedings of the National Academy of Sciences*, 115(52):E12201–E12208, 2018.

[4] R. W. Floyd. Algorithm 97: shortest path. *Commun. ACM*, 5:345, 1962.

[5] Z. Li1, T. Ren, X. Ma, S. Liu, Y. Zhang, and T. Zhou. Identifying influential spreaders by gravity model. *Scientific Reports*, 9:8387, 2019.

[6] L. L. Ma, C. Ma, H. F. Zhang, and B. H. Wang. Identifying influential spreaders in complex networks based on gravity formula. *Physica A*, 451:205–212, 2015.

[7] H. Mahmoud and A. Chulahwat. Unraveling the complexity of wildland urban interface fires. *Scientific Reports*, 8:9315, 2018.
